# Transposon clusters as substrates for aberrant splice-site activation

**DOI:** 10.1080/15476286.2020.1805909

**Published:** 2020-09-23

**Authors:** Maria Elena Vilar Alvarez, Martin Chivers, Ivana Borovska, Steven Monger, Eleni Giannoulatou, Jana Kralovicova, Igor Vorechovsky

**Affiliations:** a School of Medicine, University of Southampton, Southampton, UK; b Slovak Academy of Sciences, Institute of Molecular Physiology and Genetics, Bratislava, Slovak Republic; c Computational Genomics Laboratory, Victor Chang Cardiac Research Institute, Darlinghurst, Australia; d St. Vincent’s Clinical School, University of New South Wales, Sydney, Australia

**Keywords:** Transposed element, RNA processing, genetic disease, mutation, splice site, lariat branch point, RNA secondary structure, DBASS3, DBASS5

## Abstract

Transposed elements (TEs) have dramatically shaped evolution of the exon-intron structure and significantly contributed to morbidity, but how recent TE invasions into older TEs cooperate in generating new coding sequences is poorly understood. Employing an updated repository of new exon-intron boundaries induced by pathogenic mutations, termed DBASS, here we identify novel TE clusters that facilitated exon selection. To explore the extent to which such TE exons maintain RNA secondary structure of their progenitors, we carried out structural studies with a composite exon that was derived from a long terminal repeat (LTR78) and *Alu*J and was activated by a C > T mutation optimizing the 5ʹ splice site. Using a combination of SHAPE, DMS and enzymatic probing, we show that the disease-causing mutation disrupted a conserved *Alu*J stem that evolved from helix 3.3 (or 5b) of 7SL RNA, liberating a primordial GC 5ʹ splice site from the paired conformation for interactions with the spliceosome. The mutation also reduced flexibility of conserved residues in adjacent exon-derived loops of the central *Alu* hairpin, revealing a cross-talk between traditional and auxilliary splicing motifs that evolved from opposite termini of 7SL RNA and were approximated by Watson-Crick base-pairing already in organisms without spliceosomal introns. We also identify existing *Alu* exons activated by the same RNA rearrangement. Collectively, these results provide valuable TE exon models for studying formation and kinetics of pre-mRNA building blocks required for splice-site selection and will be useful for fine-tuning auxilliary splicing motifs and exon and intron size constraints that govern aberrant splice-site activation.

## Introduction

Splicing removes intervening sequences or introns from eukaryotic precursor messenger RNAs (pre-mRNA) and joins consecutive or alternative exons together, generating one or more mature transcripts from a single gene [1]. Intron removal is executed by spliceosomes, large and dynamic ribonucleoprotein complexes that assemble *ad hoc* on each intron and recognize exon-intron boundaries in the pre-mRNA with a single-nucleotide precision [2]. Apart from conserved traditional signals [5ʹ and 3ʹ splice site (5ʹ and 3’ss), polypyrimidine tract (PPT), and lariat branch point sequence (BPS)], accurate selection of exon junctions requires numerous auxiliary elements, known as splicing enhancers or silencers [3–6]. These pre-mRNA motifs tend to be single-stranded [7], however, their exact structural correlates during and after transcription remain poorly understood.

Mutations or variants anywhere in exons or introns can alter splice-site selection and lead to genetic disease [8]. The most frequent outcome of archetypal ‘splicing’ mutations is skipping of one or more exons, activation of one or more aberrant splice sites, or both [9–11]. Splicing mutations can also give rise to cryptic exons (or pseudoexons) by ‘exonizing’ internal intronic sequences, often in transposed elements (TEs). TEs are repetitive sequences capable of copying themselves from one chromosomal location to another, occupying a half of the human genome [12]. Disease-causing exonizations have been found for each TE family, including long and short interspersed elements (LINEs and SINEs), retrovirus-like sequences and DNA transposons [13]. SINEs, especially abundant *Alu* elements, have been major contributors to TE exonization during evolution and in human genetic disease [13–16]. *Alu*s contain a number of decoy splice-site motifs that are readily recognized by the spliceosome, generating a substantive pool of low-inclusion exons that are likely to play an important regulatory role in quantitative gene control by targeting RNAs for nonsense-mediated decay [15,17,18]. However, the exact RNA rearrangements supporting their massive exonization potential remain obscure and structural requirements underlying their huge evolutionary success are not well understood.

Over the last decades, it has become increasingly apparent that *in silico* prediction of mutations that affect pre-mRNA processing is unsatisfactory, despite a growing number of predictive algorithms, totalling to over a hundred to date. Their poor performance stems from our limited understanding of how exactly the spliceosome selects authentic splice sites in a large excess of very similar pre-mRNA motifs that are never used, termed decoy splice sites. Splicing enhancers and silencers represent the weakest link in the puzzle: despite numerous and systematic attempts to define these motifs [6,19,20], exonic and intronic mutations in these elements often do not behave as predicted. Depending on the sequence context, the same auxilliary motifs may activate or inhibit splicing [20], thus reducing the discriminatory power of *ab initio* methods. A key obstacle is our limited insight into co- and post-transcriptional pre-mRNA folding and its dynamics that may expose, hide, approximate or separate residues involved in interactions with numerous spliceosome components [21,22]. Despite the development of transcriptome-wide RNA structural probing in the last decade [23–25], our knowledge of pre-mRNA structural motifs that govern mammalian splice-site selection remains rudimentary, with more detailed studies carried out only with a small number of exons [26–29]. Because SINEs and other TEs evolved from more structured and highly conserved RNA progenitors, such as *Alu*s from 7SL RNA [30], disease-causing TE exonizations should provide useful models to understand the requirements for cross-exon pre-mRNA folding in splice-site recognition during evolution.

The aim of our present study was to identify new mutation-induced TE exons activated in genetic disease. We have found new exonized TE clusters, defined here as iterative genomic invasions of new TEs into existing TEs. We have selected one such composite exon for RNA structural probing. We show that a point mutation activating a new 5’ss in the *F8* gene altered accessibility of splicing regulatory motifs in the *Alu*J-derived portion of the exon and of the 5’ss itself. The mutation disrupted a conserved *Alu*J stem that evolved from the central helix of 7SL RNA, liberating the optimized 5’ss from the highly conserved double-stranded conformation for interactions with spliceosomal components. The stem harbours a primordial GC 5’ss that was base-paired together with prospective exonic splicing regulatory motifs in the opposite strand for ~2 billion years. We also identify existing *Alu* exons activated by the same RNA rearrangement.

## Materials and methods

### Update of DBASS3 and DBASS5

We first updated previously developed databases of aberrant 3ʹ and 5’ss, termed DBASS3 and DBASS5 [31]. They serve as retrieval and submission tools for published disease-causing and mutation-induced aberrant splice sites that were characterized at a single-nucleotide level. Aberrant splice sites are defined here as new exon-intron boundaries induced by mutations within the authentic 5’ss (MAG/GURAGU, where/is the boundary, M is A or C and R is purine) or 3’ss (YAG/G, where Y is pyrimidine) consensus (cryptic sites), or outside these motifs (‘*de novo*’ sites) [9,32]. Although this binary classification is not strictly mutually exclusive, particularly for aberrant 3’ss, it helps us understand their location and distribution within exons and introns upon mutation [9,10,32]. Reports of aberrant splice sites published between January 2011 and December 2019 were identified through PubMed queries defined previously [31]. The search was restricted to pathogenic mutations in human disease genes that were causally associated with sequenced aberrant transcripts, typically obtained from total RNAs extracted from patients’ blood. Aberrant transcripts detected by *ex vivo* minigene studies were also included because they usually recapitulate *in vivo* splicing defects with high accuracy [33]. Exon skipping or full intron retention events where no new intron-exon boundaries were created upon mutation were not recorded.

Qualifying reports of aberrant splice sites were verified against reference sequences from GenBank (http://www.ncbi.nlm.nih.gov/Genbank) [34] and Ensembl (http://www.ensembl.org) [35]. In-house scripts (File S1, run.sh and align.py) and the NCBI BLAST+ tool were used to obtain genomic coordinates (hg38) for each aberrant splice site and underlying mutation. Coordinates were validated using Spliceogen’s in-built reference allele check [36] and any mismatches between retrieved and reference sequences were corrected manually. The intrinsic strength of aberrant splice sites and their authentic counterparts were scored using previously established models, including the Maximum Entropy (ME) Model and First-order Markov Model [37], as described previously [9,10].

### Identification of aberrant splice sites activated in TEs

Sequences surrounding validated aberrant splice sites were used as an input to search for TEs with a crossmatch search engine of RepeatMasker (v. 3.0), employing its highest sensitivity option [38]. TEs aligned with aberrant splice sites were classified as described previously [13].

### *Preparation of wild-type and mutated* F8 *transcripts*

To support computational predictions [39,40] of pre-mRNA secondary structures across the TE *F8* pseudoexon, we synthesized wild-type (WT) and mutated (*F8* c.5998 + 530 C > T) RNAs for structural probing. Probe templates were prepared using nested PCR with outer primers (F8-F, 5ʹ-TGT CAC AGT ACT TTC CTA GGG A; F8-R, 5ʹ- TGG CAC TTT CAT AGC TCA CTG) and probe- and mutation-specific inner primers (F8T7-F, 5ʹ-TAA TAC GAC TCA CTA TAG GGA GAG GCC TTC GGG CCA AAA TAG ATT TGG CCA GGT GC and F8-R, 5ʹ-GAA CCG GAC CGA AGC CCG ATT TGG ATC CGG CGA ACC GGA TCG AGG TCT T[G/A]C TTT GTC ACC CA; where the two alleles are separated by a slash in square brackets; linkers are underlined). The linkers allow the reverse transcriptase (RT) to become fully processive prior to reaching the region of structural interest and also prevent non-templated primer extension products from masking structural information [41]. The forward primer also contained a T7 promoter sequence. PCR products were purified using GeneJET Gel Extraction Kit (ThermoFisher) and Sanger-sequenced to confirm the desired mutation. The 181-nucleotide RNA probes were transcribed using MEGAscript™ T7 Transcription Kit (Invitrogen) according to manufacturer’s recommendations. Transcripts were purified using TRI-Reagent (Invitrogen) and quantified with UV-spectroscopy. Their integrity was confirmed on a 8.3 M urea-8% polyacrylamide gel.

### RNA structural probing

Validated transcripts were denatured at 95 °C for 90 seconds and cooled to 4 °C. An equal volume of a 2x reaction buffer was added to 10 pmol of each RNA probe to a final concentration of 100 mM KCl, 40 mM HEPES (pH 7.5) and 0.5 mM MgCl_2_. The samples were incubated at 37 °C for 45 min. 2-methylnicotinic acid imidazolide (NAI) or dimethyl sulphate (DMS) were added to a final concentration of 100 mM and allowed to react with the RNA for 5 min at 37 °C (NAI) or for 4 min at room temperature (DMS). The reaction was quenched with a freshly prepared dithiothreitol at a final concentration of 0.2 M (NAI) or 0.5 M (DMS) and mixed thoroughly. The reactions were immediately loaded on to the RNA Clean&Concentrator™-5 (ZYMO Research). RNAs were eluted in 10 µl of RNase-free double-distilled water and 6 µl of purified RNA was mixed with 1 µl of a 5 µM solution of the Cy5-labelled universal primer (5ʹ-GAA CCG GAC CGA AGC CCG). The samples were heated at 75 °C for 3 min. Two µl of 5x RT reaction buffer were added to each sample to a final concentration of 50 mM Tris-HCl (pH 8.3), 75 mM KCl, 3 mM MgCl_2_, and 5 mM dithiothreitol. The reaction was incubated at 35 °C for 5 min, which was followed by the addition of 0.5 µl of dNTPs (10 mM) and 0.5 µl of Superscript III RT (200 U/µl), and a 15-min incubation at 50 °C. Next, 0.5 µl of 2 M NaOH was mixed with each RT reaction and samples were heated at 95 °C for 15 min to degrade RNA and denature RT. The reaction was then mixed with an equal volume of 2x stop solution, containing 95% deionized formamide, 20 mM Tris (pH 7.5), 20 mM EDTA (pH 8.0) and Orange G (Abcam) for tracking. The samples were heated at 95 °C for 5 min and the RT products were size-fractionated on 8.3 M urea-8% polyacrylamide gels at the constant power of 65 W for 3–5 hrs. Gel images were collected with a Typhoon PhosphorImager 9210 and individual bands were quantified using ImageQuant 8.2. The nucleotide identity of RT stops was determined from dideoxy-sequencing lanes run in parallel. DMS and NAI signals were normalized to the fully extended product [42] or using the 2/8 rule [43]. Signals from negative controls were subtracted from DMS+ and NAI+ reactions. RNA secondary structure predictions were carried out with or without constraints of selective 2ʹ-hydroxyl acylation analysed by primer extension (SHAPE) NAI data using the Vienna cluster or RNAstructure [41,44,45]. The PU (probability of unpaired) values were computed as described [7] using the WT and mutated *F8* pseudoexon and 100-nt flanking intron sequences as an input. PU values predict single-stranded conformation of auxiliary splicing motifs and were defined previously [7].

For enzymatic probing, we digested the same probes with RNAse A (Ambion), which cleaves single-stranded pyrimidines. The reactions were incubated in a final volume of 100 μl at room temperature for 3 min. A control aliquot of RNA without RNase A was processed simultaneously with digested samples. Reactions were stopped by adding SDS (0.5%) and proteinase K (200 μg/μl) and incubated at 55°C for 1 hr. The cleaved RNA was purified using TRI-Reagent and 200 µg of each probe was reversed transcribed as described above. Signals from digested products were quantified using ImageQuant 8.2 and normalized to the full-length signal.

## Results

### Distribution of aberrant splice sites in updated DBASS

DBASS3 and DBASS5 show sequences of 1,074 experimentally verified *de novo* or cryptic splice sites that were activated by disease-causing mutations in ~390 genes. Full DBASS data are freely available at http://www.dbass.org.uk or http://dbass.soton.ac.uk, with direct links to DBASS3 or DBASS5 at http://dbass3.soton.ac.uk and http://dbass5.soton.ac.uk. DBASS3 currently holds 381 aberrant 3’ss in 193 genes that were causally associated with ~200 distinct human phenotypes. DBASS5 provides details of 693 aberrant 5’ss that were activated in 283 disease genes and were responsible for ~290 recognizable phenotypes.

**Figure 1.**
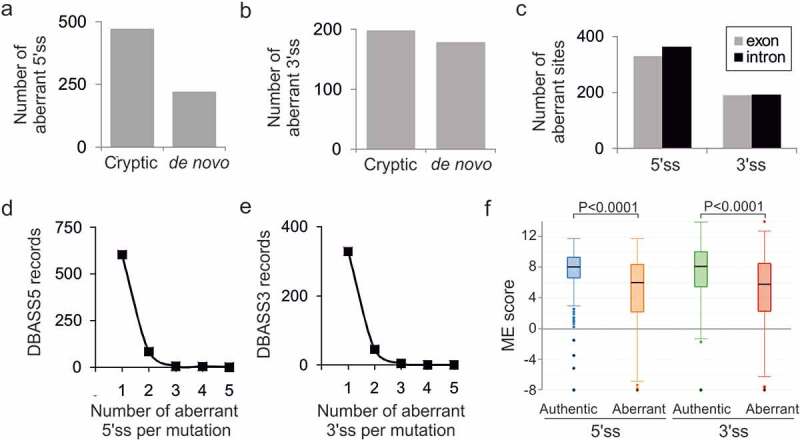
Characteristics of mutation-induced aberrant 3ʹ and 5ʹ splice sites that resulted in genetic disease. (a, b) Total number of cryptic and *de novo* 5’ss (a) and 3’ss (b) in DBASS. (c) Location of aberrant splice sites. (d, e) Proportion of multiple aberrant 5’ss (d) and 3’ss (e) activated by a single mutation. (f) The intrinsic strength of aberrant 5ʹ and 3’ss and their authentic counterparts. Their numbers are shown in panels (a) and (b). Whiskers/boxes denote quartiles, black lines denote medians. P values for the indicated comparisons of mean ME scores were derived by unpaired t-tests. T-values were 13.01 (5’ss) and 7.85 (3’ss)

Breakdown of the updated DBASS data showed that *de novo* splice sites were more frequent among aberrant 3’ss than among aberrant 5’ss (Figure 1(a,b); P < 0.05, χ^2^ test). We attribute this bias mainly to the accumulation of *de novo* 3’ss upstream of authentic counterparts in extended 3’ss motifs that are located in the AG-dinucleotide exclusion zone and define the first splicing step, *ie*. PPT and BPS [9]. In addition, the short conserved 3’ss consensus (YAG/G) required for the second step of splicing may be more easily created by point mutation than the longer 5’ss motif. However, the higher fraction of *de novo* 3’ss in PPT upstream of authentic counterparts [9] is offset by an increased frequency of cryptic 3’ss downstream of authentic sites than in the upstream region where the AG dinucleotides are depleted. As a result, the overall distribution of aberrant 3’ss and 5’ss in introns and exons is similar (Figure 1(c)).

The fraction of multiple aberrant 5’ss activated by a single pathogenic mutation was ~13% (90/693 cases, Figure 1(d)). This percentage was very similar for aberrant 3’ss (Figure 1(e), 49/381; P > 0.05, χ^2^ test), indicating that multiple aberrant 3ʹ and 5’ss are induced by disease-causing mutations with approximately equal frequencies.

### Intron and exon size constraints that hold back strong contenders

Both initial [9,10] and updated (Figure 1(f)) DBASS records showed that although aberrant splice sites were on average significantly weaker than their wild-type authentic counterparts, this was not always the case. Updated DBASS data indicated that ~19% of cryptic 3’ss and 13% cryptic 5’ss were intrinsically stronger than their wild-type canonical partners, yet these strong cryptic sites were used only if the authentic 5ʹ or 3’ss consensus motifs were inactivated or weakened by mutation (Table S1). The median ME score of these cryptic sites was higher by 1.51 (3’ss) or 1.17 (5’ss) than their weaker canonical competitors (P < 0.001, t-tests).

**Figure 2.**
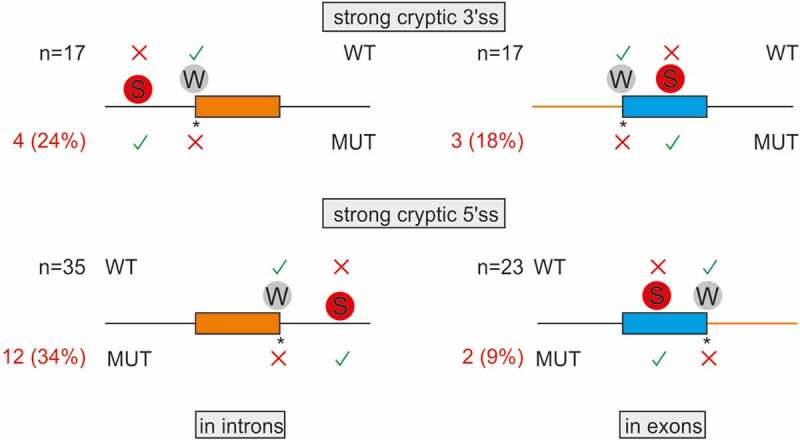
Intron and exon size constraints that hold back strong contenders. Location of 92 pairs of strong-cryptic (S) and weak-authentic (W) splice sites in introns (left) and exons (right). Their location is denoted by circles. For each pair, the ME score of cryptic site was higher than for its authentic counterpart (Table S1). Activation of each cryptic site resulted in human genetic disease (Table S1). Introns are denoted by horizontal lines, exons by boxes. WT, MUT; wild-type and mutated splice sites, respectively. Asterisk, mutation; X, splice-site repression; √, splice-site activation. The total number of S-W pairs in each group (N) is shown to the left. The number of aberrant 3’ss (top) or aberrant 5’ss (bottom) with small (≤100 nt) adjacent exons (left) or small (≤200 nt) adjacent introns (right) is in red; their proportions are in parentheses. Size-restricted segments are in orange

Employing a sample of 92 pairs of weak-authentic and strong-cryptic splice sites, we explored if their activation was constrained by the length of adjacent introns or exons (Table S1). The breakdown of DBASS entries showed that introns with over a fifth of such strong cryptic 3’ss were flanked by small (≤100 nt) exons downstream (Figure 2, *top left*). These cases are exemplified by reports of aberrant 3’ss in *IVD* [46], *IDS* [47] or *GCK* [48] genes. Smaller exons harbour on average less decoy sites than larger exons and are also generally less efficiently recognized by the spliceosome, which may enforce activation of strong cryptic 3’ss in the upstream intron. Similarly, a comparable fraction of strong cryptic 3’ss activated in exons had very small (<200 nt) neighbouring introns (Figure 2, *top right*). Such introns may lack 3’ss consensus motifs, are recognized by intron definition rather than exon definition, and may require cross-intron bridging interactions [49–51], which might force the spliceosome to select a new 3’ss in the downstream exon(s). Analogous size constraints were found for cryptic 5’ss (Figure 2, *bottom*): a third of strong cryptic 5’ss activated in introns had small upstream exons, as exemplified by intronic 5’ss reported in *ATM* [52], *COL7A1* [53], and *COL1A1* [54]. This fraction (34%) appeared to be larger than ~9% exonic cryptic 5’ss with small downstream introns (P = 0.02, Fisher’s exact test; Figure 2, *cf. bottom left and right*).

Recognition of cryptic 3ʹ or 5’ss in introns can also leave the remaining intronic portions too short to be spliced out effectively. If the residual part is near or below the minimum size of canonical human introns, estimated at ~70 nt [55], exon skipping may ensue or the spliceosome may select another site elsewhere. For example, mutation of a weak 5’ss in the *RB* gene activated a strong cryptic 5’ss downstream, but the remaining intronic portion was only 35 nt, which led to skipping of the downstream exon [56]. Activation of cryptic sites in very short exons does occur but we found only two examples in the literature [57,58], suggesting that these cases are very rare.

To further explore the importance of size limits, we computed median exon sizes for strong-aberrant 3ʹ or 5’ss activated in introns. The median of their adjacent exons was somewhat shorter than the median size of human internal exons (127 and 125 nt for 3 and 5’ss, respectively, *versus* 139 nt) but was longer than the median of human exons that were skipped as a result of disease gene mutations instead of activating aberrant sites (111 nt) [59].

Together, these data demonstrate intron and exon size constraints for about a fifth of strong-cryptic counterparts of weak-authentic sites and highlight the importance of incorporating size limits into *in silico* prediction tools.

### How TEs combine forces to activate mutation-induced aberrant splice sites

Consistent with data in Figure 1(a,b), the number of pseudoexons activated via 5’ss was higher than those activated via 3’ss (~18% versus ~5%, *P*< 10^−6^). We also found more TEs in the former than the latter pseudoexons (*cf*. Tables 1 and 2). Closer examination of these cases uncovered pseudoexons that were supported by TE clusters. They are discussed below.

**Table 1.** Summary of TEs detected in new DBASS3 records

TE superfamily	TE family	Gene	Phenotype	Reference
SINE	*Alu*Y	*NSUN2*	Dubowitz syndrome	[104]
DNA/LINE	MER58A (3’ss)/L1 (5’ss)	*COL4A5*	Alport syndrome	[63]
LTR/SINE	LTR78 (3’ss)/*Alu*J (5’ss)	*F8*	Haemophilia A	[62]
LINE	L2c-3ʹend	*GLA*	Fabry disease	[105]

**Table 2.** Summary of TEs detected in new DBASS5 records

TE superfamily	TE family	Gene	Phenotype	Reference
DNA	Charlie1a/DNA	*BRCA2*	Breast cancer	[106]
LINE	L2b_3end	*NF2*	Neurofibromatosis type 2	[107]
SINE	*Alu*S	*ATM*	Ataxia-telangiectasia	[108]
LTR	MER20	*PKD1*	Autosomal dominant polycystic kidney disease	[109]
DNA	Tigger2a	*CFTR*	Cystic fibrosis	[110]
LTR/SINE	LTR78 (3’ss)/*Alu*J (5’ss)	*F8*	Haemophilia A	[62]
SINE	*Alu*Sx	*BRCA1*	Early onset breast and ovarian cancer	[111]
SINE	MIR3	*DMD*	Becker muscular dystrophy	[112]
LINE	L1MD3-3end (5ʹ end of BPS)	*GPR143*	Ocular albinism type 1	[113]
SINE	*Alu*Sx	*VPS4B*	Dentin dysplasia I	[114]
LTR	MER31A	*CSTB*	Unverricht-Lundborg disease	[115]
LTR	THE1-int	*MSH2*	Lynch syndrome	[116]
SINE	MIRb	*CYP17A1*	17α-hydroxylase deficiency	[117]
SINE	*Alu*Y (3’ss and PPT/BPS)	*CEP290*	Leber congenital amaurosis	[118]
LINE	MER58A (3’ss)/L1 (5’ss)	*COL4A5*	Alport syndrome	[63]

**Figure 3.**
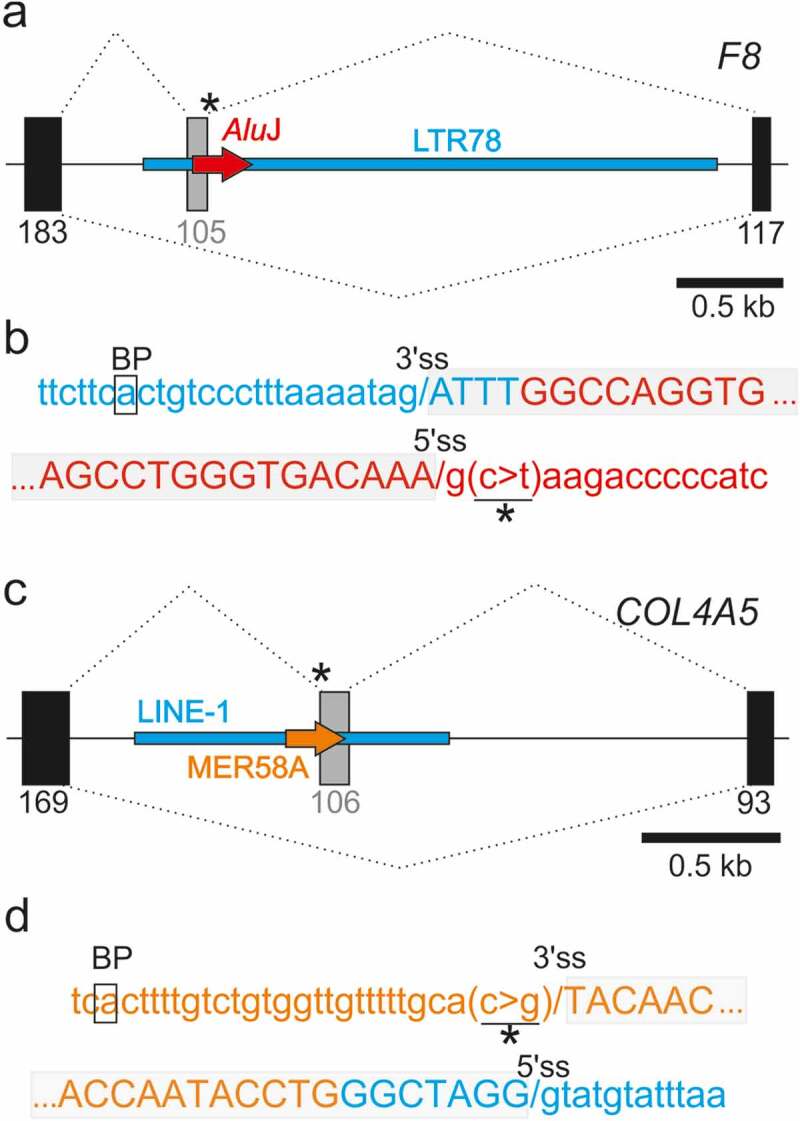
New TE clusters as substrates for aberrant splice site activation. (a,b) Mutation-induced exonization of the LTR78-*Alu*J cluster in *F8* intron 18. (a) Schematics of mutation-induced pseudoexon splice sites located in separate TEs. Canonical exons are denoted by black boxes, pseudoexon by a grey box. Exon length is in nucleotides below. A scale at the bottom is in kilobases (kb). Mutation (asterisk) activated the pseudoexon via a *de novo* 5’ss, leading to mild haemophilia A [62]. Dotted lines above and below the pre-mRNA indicate aberrant and canonical splicing, respectively. (b) Sequences around pseudoexon splice sites. Splice sites are denoted by a slash. A putative branch point adenine in LTR78 (boxed) was predicted by the SVM-BP algorithm [73]; the disease-causing mutation is underlined. Sequences in blue and red are derived from LTR78 and *Alu*J, respectively. (c,d) Mutation-induced exonization of a LINE and MER58A cluster in *COL4A5*. (c) Schematics of mutation-induced splice sites activated in separate TEs. For full legend, see panel (a). (d) Sequences around pseudoexon splice sites. Sequences in blue are derived from a LINE-1 copy, sequences in orange from a MER58A copy. Mutation (asterisk) creating the 3’ss AG led to pseudoexon activation, causing Alport syndrome [63]

Figure 3(a) shows activation of a pseudoexon 3’ss in an antisense long terminal repeat (LTR) element upon single-nucleotide substitution creating a *de novo* 5’ss in the left arm of a sense *Alu*J in *F8* intron 18. The *Alu*J copy was retroposed into a more ancient LTR78 and contributed the 5’ss and most of the pseudoexon seqence (Figure 3(b)). Alignments of the LTR78-derived 3’ss/BPS of the pseudoexon to a set of previously reported LTR exons [60,61] failed to identify any existing LTR exon that had 3’ss activated at the same LTR position. This suggests that the selection of entirely new LTR-derived 3’ss and the BPS have been assisted by a combination of two TE families (LTR and SINE), one contributing the BPS/PPT/3’ss motifs and the other providing the 5’ss. To our knowledge, this case is also the first LTR-derived cryptic exon in the *F8* gene. The affected individual had a mild haemophilia [62], which does not appear to limit reproductive fitness, leading to fixation of the new exon-producing allele in the population.

**Figure 4.**
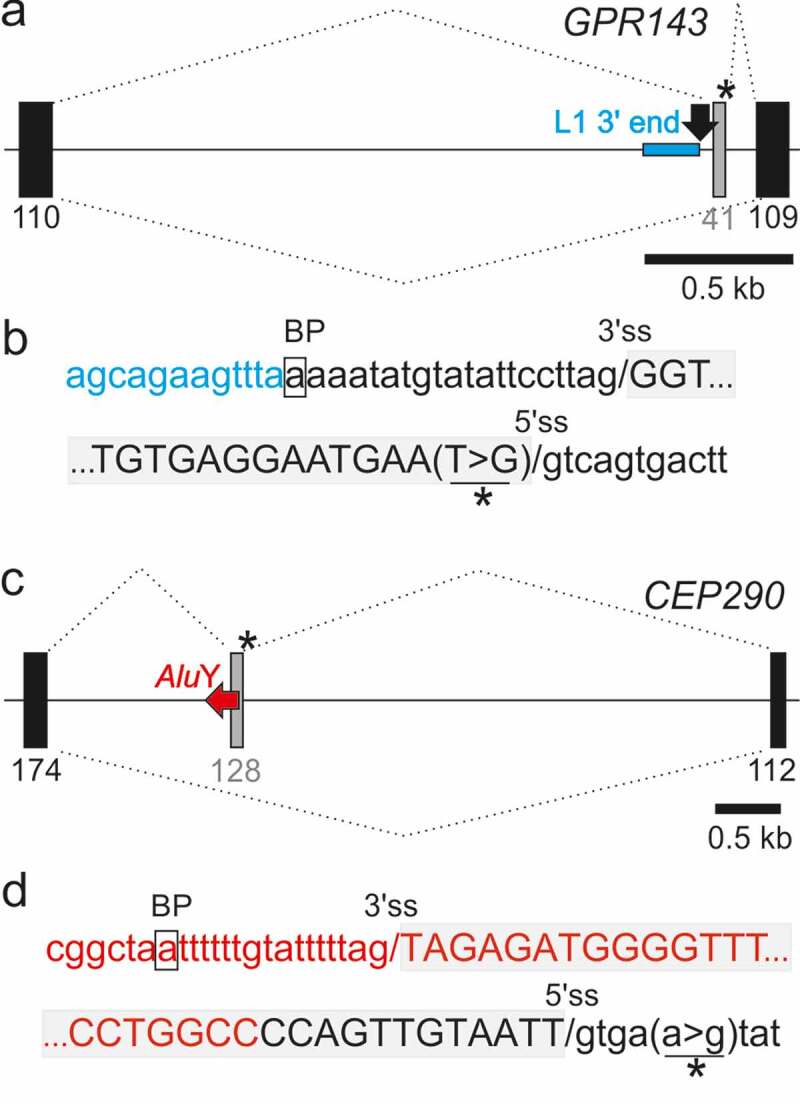
TEs can contribute only a portion of traditional splicing elements and can be activated by distant mutations. (a,b) A LINE fragment contributing the 5ʹ part of the predicted branch point sequence of the *GPR143* pseudoexon. (a) Schematics of the mutation-induced pseudoexon. For full legend, see Figure 3(a). Blue horizontal rectangle denotes the the 3ʹ end of L1MD3. Black arrow denotes BPS. (b) Sequences around pseudoexon splice sites. (c,d) *Alu*Y as a source of the BPS, PPT and 3’ss of a *CEP290* pseudoexon. (c) Schematics of the mutation-induced pseudoexon. Horizontal red arrow denotes a full-length *Alu*Y copy. For full legend, see Fig. 3A. (d) Sequences around pseudoexon splice sites

Figure 3(c) shows that the 3’ss of a *COL4A5* pseudoexon, including the predicted BPS, was derived from a DNA transposon (MER58A) that was inserted into a more ancient long interspersed element (LINE1 or L1). The L1 copy contributed the full 5’ss consensus and the 3ʹ end of the pseudoexon (Figure 3(d)). As with the *F8* pseudoxon [62], the mild Alport syndrome was associated with <100% utilization of aberrant splice sites in mature transcripts in some tissues [63]. The syndrome can manifest as a late-onset condition [64], also without reducing reproductive fitness of affected males. Finally, Figure 4(a-b) illustrate that a recognizable TE can contribute only the 5ʹ end of the predicted BPS but neither 3’ss nor 5’ss while Figure 4(c-d) panels show an example of antisense *Alu* exonization as a result of a downstream mutation outside this element.

### Crosstalk between traditional and auxiliary splicing signals that evolved from opposite 7SL RNA termini

**Figure 5.**
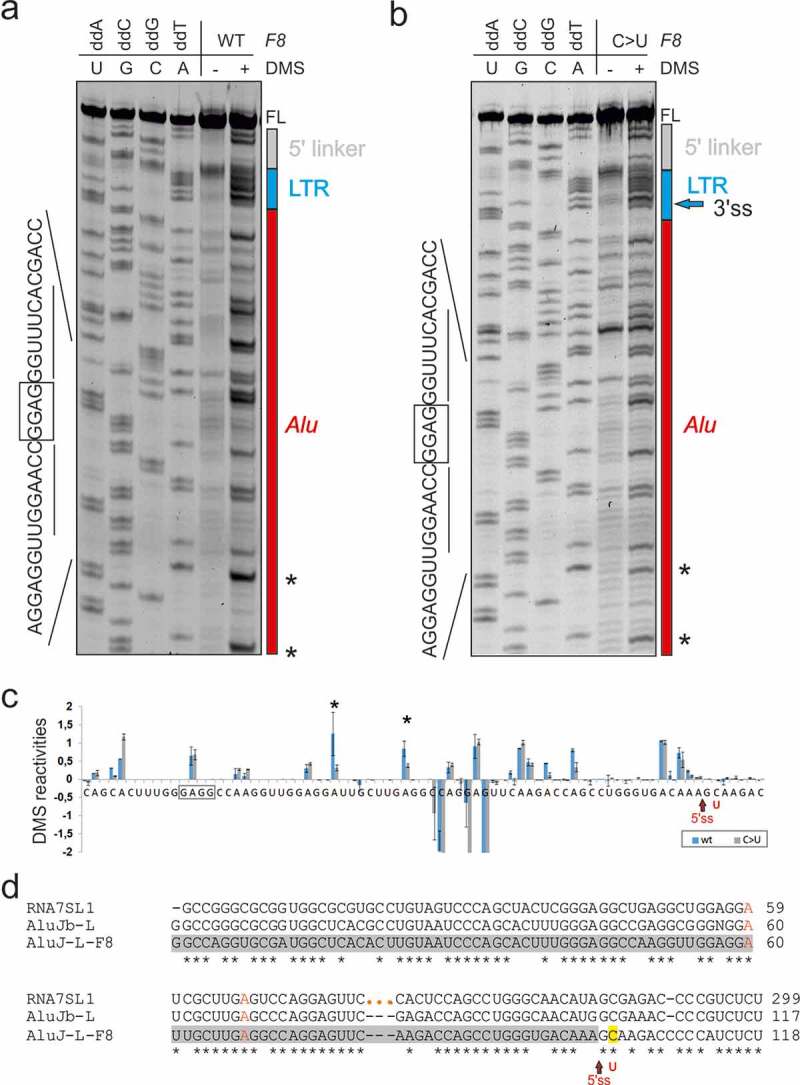
Structural probing of a composite LTR78/*AluJ* exon activated in the *F8* gene. (a,b) Denaturing polyacrylamide gels with a dideoxynucleoside triphosphate-generated stops (left) run in parallel with primer extension reactions for the WT (a) and mutant (b) *F8* probes in the presence (+) and absence (-) of DMS. FL, full-length transcript. Predicted *Alu*J-derived apical tetraloop/stem is boxed/underlined. (c) Normalized DMS reactivities for WT and mutant *F8* RNAs. Columns are means and error bars are SDs, as calculated from 2 independent experiments. Asterisks denote significant decline of DMS reactivities in the mutant (P < 0.05, ANOVA with Tukey’s post-hoc test); negative values were cut off at −2. The mutated residue (*F8* c.5998 + 530 C > T) that activated an intronic 5’ss and caused haemophilia [62] is in red. (d) Sequence alignment of the human 7SL RNA gene *(RNA7SL1*), Repbase *Alu*Jb consensus [102] and the exonized left arm of the *Alu*J copy in *F8* intron 18. Three orange dots separate the 5ʹ (1–80) and 3ʹ (262–299) termini of 7SL RNA that gave rise to mammalian free left *Alu* monomers [30,66]. Conserved adenines with differential DMS reactivities in the exonized left arm of the *F8 Alu*J are in red. The pseudoexon is highlighted in grey. The exonized left arm of *F8 Alu*J and corresponding 7SL RNA sequences are ~70% identical

**Figure 6.**
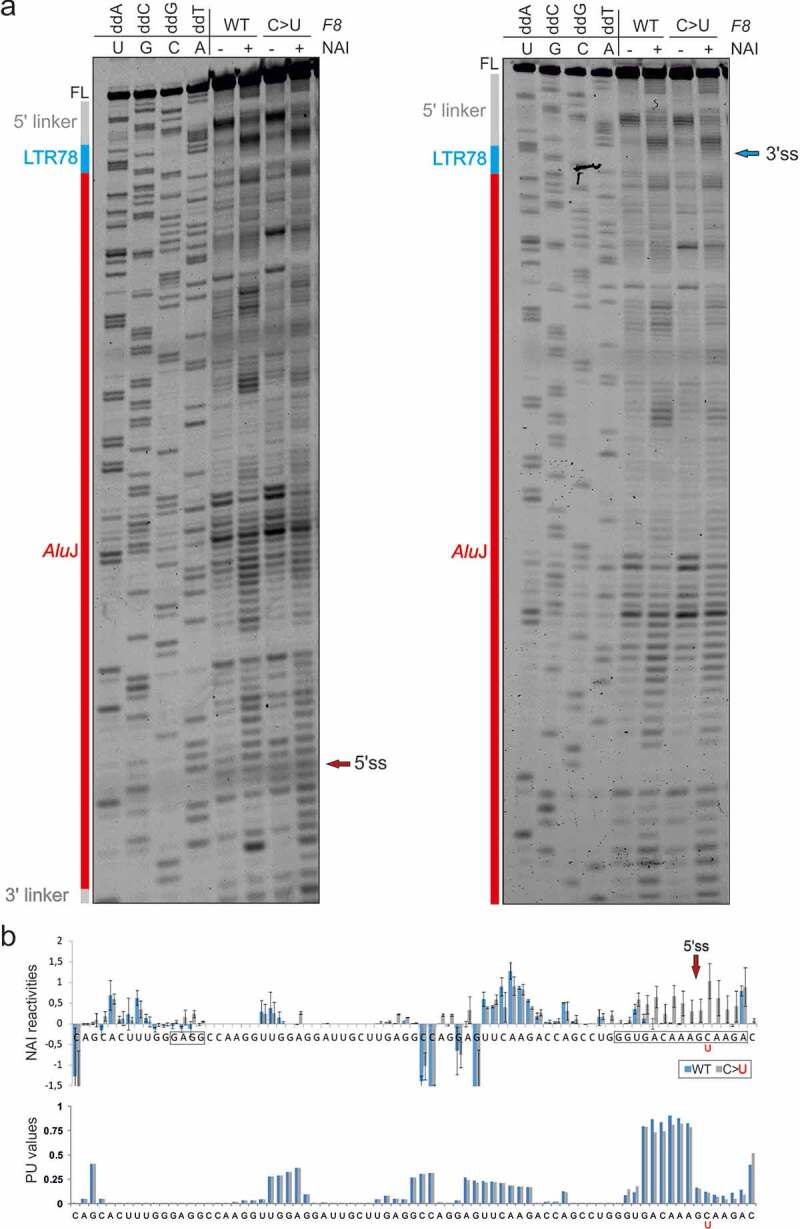
SHAPE reactivities for WT and mutated *F8* transcripts. (a) SHAPE gels with NAI-modified WT and mutant RNA probes that visualize their 3ʹ (*left panel*) and 5ʹ (*right panel*) portions. (b) Normalized NAI reactivities (*upper panel*) and PU values (*lower panel*) for identical RNA segments

**Figure 7.**
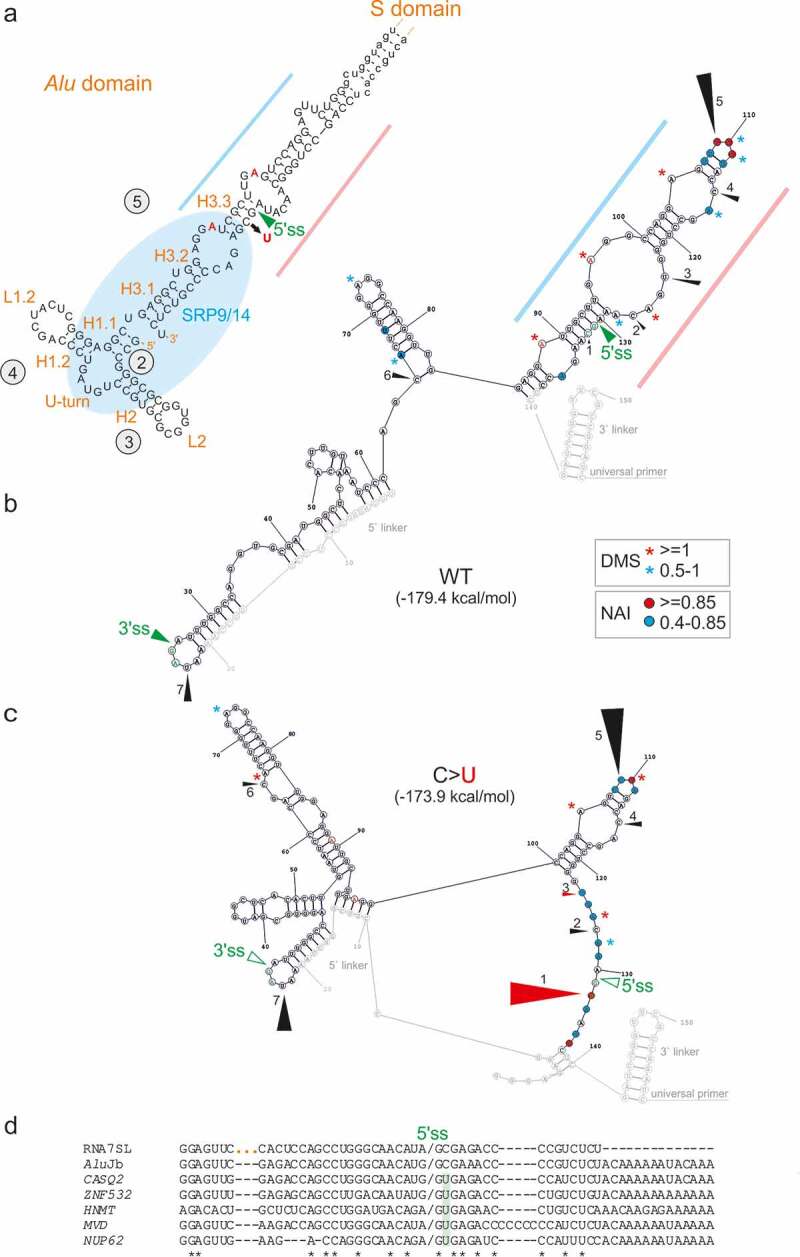
Comparison of the secondary structure of 7SL RNA within the *Alu* domain of SRP and SHAPE-guided structures of WT and mutated LTR78/*Alu*J RNAs. (a) Secondary structure of 7SL RNA within the *Alu* domain of SRP [65,66,99]. Helices (H) and loops (L) in orange are numbered according to a previously published topology [99]. The central stem (H3) is also known as helix 5 in the unifying nomenclature of all SRPs (circled) [67], stem V [68] or stem III [65]. Lower case letters indicate RNA sequences that are absent in the left *Alu* arm consensus [65] (Figure 5(d)). The C > U mutation is denoted by a black arrow. Blue and pink rectangles denote the central stem portions that are most similar to the *F8 Alu*J structure and were derived from the 5ʹ and 3ʹ parts of 7SL RNA, respectively. Conserved adenines with differential DMS reactivities in WT and mutated *F8* probes are in red. (b,c) SHAPE-guided secondary structure of the WT (b) and mutant (c) LTR78/*Alu*J RNAs. Normalized DMS and NAI reactivities (colour coded as indicated) are shown in Figs. 5 and 6. Black triangles indicate major RNase A cleavage sites (numbered 1–7); their size indicates normalized cleavage intensities in the WT (Fig. S1). In the mutant (c), triangle sizes indicate changes in relative cleavage intensities as compared to the WT; red triangles denote >3-fold differences between the mutant and WT. The linkers and RT primers are highlighted in grey. Alternative structures are shown in Fig. S2. Decoy and active 5’ss are marked by closed and open green triangles, respectively. Secondary structures in panels (b) and (c) were predicted by RNAstructure [103] using normalized NAI constraints and default options. d Alignment of existing sense *Alu*J exons that employ the same decoy 5’ss as the *F8 Alu*J copy. The 5’ss are denoted by a slash. Dashes are alignment gaps; three orange dots separate the 5ʹ and 3ʹ parts of 7SL RNA that gave rise to *Alu-*like elements [30,66]. Full sequences of sense *Alu*s that use 5’ss homologous to that activated in the *Alu*J copy in *F8* are in Table S2. Their alignment is in Figure S3

We selected the LTR78/*Alu*J exon for structural probing of *F8* transcripts representing the splicing-proficient mutant and splicing-deficient WT (Figures 3(a,b), 5–7, S1, and S2). To prepare the probes from a repeat-containing DNA template without the patient’s material, we first employed PCR primers that amplify a larger and unique *F8* intron 18 segment containing the LTR78/*AluJ* cluster. In nested reactions, we used probe- and allele-specific primers that included a T7 promoter and linkers. Using DMS probing, we found that two adenines were more reactive in the unspliced WT than in the splicing proficient mutant RNA (Figure 5(a-c)). Both residues are conserved and unpaired in secondary structure models of the left *Alu* arm and 7SL RNA [65] and the LTR78/*Alu*J exon (Figures 5(d) and 7). The two residues are also single-stranded in structural models of other *Alu*-like elements that evolved from 7SL RNA, including brain cytoplasmic 200 RNA (BC200) [66]. Both adenines were within predicted exonic splicing regulatory motifs (Table 3). Structural probing with NAI, which reacts with each nucleotide albeit not with the same affinity [43], revealed greater flexibility across the authentic 5’ss in the mutant than across the decoy 5’ss in the WT (Figure 6(a,b)). The increased accessibility of mutated 5’ss was confirmed by probing with RNase A, which digests unpaired pyrimidines (Fig. S1, Figure 7(b,c)). The highest normalized NAI reactivities in the WT were found for the apical CAA triloop that caps the central *Alu*J stem instead of the 7SL RNA moiety of the SRP S-domain (Figures 6 and 7). The triloop has strong predicted enhancer activities (Table 4) and is maintained in the mutant (Figures 6 and 7), suggesting that it promotes inclusion of the composite exon in mature transcripts or may even be required for high-inclusion *Alu*J exonizations.

**Table 3.** Genomic context of adenines with differential DMS reactivities between WT and mutant *F8.*

Hexamers around A60^1^	ESRseq score[2]	Assignment	Hexamers around A68^1^	ESRseq score[2]	Assignment
GGAGGA	0.41	Enhancer	GCTTGA	0	Neutral
GAGGAT	0.53	Enhancer	CTTGAG	0	Neutral
AGGATT	0	Neutral	TTGAGG	−0.33	Silencer
GGATTG	0.24	Enhancer	TGAGGC	0	Neutral
GATTGC	0.12	Enhancer	GAGGCC	0.38	Enhancer
ATTGCT	−0.11	Silencer	AGGCCA	0	Neutral

^1^Adenines are numbered as in Figure 5(d). [2]ESRseq scores and assignments were as defined by Ke and co-workers [6].

**Table 4.** Enhancer activities of the SHAPE-predicted triloop

Hexamer	ESRseq score[1]	Assignment
GTTCAA	0.22	Enhancer
TTCAAG[2]	0.26	Enhancer
TCAAGA[2]	0.46	Enhancer
CAAGAC	0.71	Enhancer

The triloop in the *F8 Alu*J copy (underlined in overlapping hexamers) caps the central stem in the RNA moiety of SRP instead of the S domain (Figure 7). [1]ESRseq scores and assignments were as defined by Ke and co-workers [6].[2]These enhancers were identified in independent studies [20,119].

Comparison of the secondary structure of 7SL RNA with the SHAPE-guided predictions incorporating DMS and enzymatic probing of *F8* transcripts revealed that the mutated cytosine was in the middle of *Alu*J-derived helical structure that evolved from helix 3.3 (H3.3; also known as helix 5b; refs. [67,68]) of the 7SL RNA central stem (Figure 7(a-c)). Although the C > U mutation introduces a G-U wobble pair in H3.3, the mutated H3.3 progeny in *F8 Alu*J was clearly destabilized (Fig. S1). The destabilization was supported by alternative secondary structures (Fig. S2). This finding suggests that the optimized 5’ss was released from the paired conformation in H3.3 to a more flexible, possibly unpaired conformation. The rearrangement may also stabilize a hairpin capped by an apical tetraloop GAGG (Figure 7(b,c)). The same tetraloop increased inclusion of a SINE-derived exon in mature transcripts [26] and is overrepresented among systematically derived splicing enhancer hexamers [6], suggesting that it promotes inclusion levels of the LTR78/*AluJ* exon in mRNAs.

To explore if the same decoy GC 5’ss was used by existing *Alu*-derived exons, we inspected the database of exonized TEs [60]. Among ~850 exonized *Alu*s in the human transcriptome, 110 were found in the sense orientation and 22 of them carried *Alu*J-derived fragments [60]. Comparisons of the 22 exons with the *F8* exon and 7SL RNA revealed at least five existing *Alu*J exons that used identical decoy 5’ss (Table S2, Figure 7(d)). Each extant *Alu*J exon contained the C > U mutation at the same 5’ss position as the LTR78/*Alu*J pseudoexon in *F8* (Figure 7(d)). We then extended our analysis to other *Alu* subfamilies and identified at least 9 *Alu*S exons, 4 *Alu*Y exons and 3 exonized free left *Alu* monomers activated via homologous 5’ss (Table S2, Fig. S3). In addition, analysis of splicing regulatory hexamers around their *F8* triloop homologs confirmed that they have largely enhancer activities, with a median ESRseq score of 0.41 (Table S3). Homologs of the CCA triloop in exons derived from younger *Alu* families were usually CGA (Fig. S3).

Taken together, the C > U mutation creating a *de novo* 5’ss of the LTR78/*AluJ* exon in *F8* altered accessibility of a composite splicing regulatory motif ~35-45 nt upstream and of the 5’ss itself. The mutation destabilized the *Alu*J ortholog of 7SL RNA H3.3 (also known as H5b) and possibly the entire central stem. The H3.3 disruption would free the highly conserved decoy 5’ss from the double-stranded conformation to allow base-pairing with the U1 small nuclear RNA, a critical interaction for selection of human 5’ss by U1 small nuclear ribonucleoproteins [69 and references therein]. The same mutation and rearrangement unleashed homologous decoy GC 5’ss in many existing intronic *Alu*s, generating almost a fifth (~19%) of sense *Alu*s exons in at least 20 other human genes (Table S3 and Fig. S3).

## Discussion

### DBASS and prediction of mutation-induced splicing errors

The impact of DNA variants or mutations on RNA processing has been notoriously difficult to accurately predict from the sequence alone without structural information. By providing large sets of verified aberrant transcripts induced by recent mutations, the updated DBASS offers an opportunity to better understand structural consequences of Nature’s own human experiments, i.e. pathogenic mutations causally associated with verified splicing defects. Our data support the notion that recently exonized TEs and their TE partners have a more predictable folding landscape than average coding sequences. Their identification thus provides a useful resource for future pre-mRNA structural studies, ultimately leading to more accurate predictions.

DBASS now contains genomic coordinates and can be better integrated into currently available predictive algorithms, including variant interpreters for clinical use, such as ClinVar [70]. DBASS3 and DBASS5 data were previously used to develop our own predictive tools, including CRYP-SKIP, which can distinguish between cryptic splice site activation and exon skipping upon mutation of 3’ss or 5’ss [59], or HOT-SKIP, which computes the splicing enhancer/silencer profile for all possible point mutations at each exon position and identifies nucleotide substitutions that are most likely to skip the exon [71]. The intron or exon size restrictions observed for a subset of DBASS records (Figure 2) suggest that intron and exon length constraints should improve predictive metrics of these and other *in silico* tools. Apart from size thresholds discussed for Figure 2, additional length limitations are likely to exist. For example, the use of aberrant splice sites could be restricted by noncanonical (distant) BPS. Such BPS reside further upstream of their usual location 19–37 nt from 3’ss, a home of 90% of human BPS [72]. Mutations creating new 3’ss downstream or upstream of distant BPS are likely to violate their large AG exclusion zones and/or compete with the use of existing splice sites [73,74]. The need to incorporate the size thresholds into predictive algorithms is consistent with a recently published superior performance of a convolutional neural network model scanning 10,000 flanking nucleotides as compared to a 80-nucleotide model [75], and with other studies that adopted length constraints albeit without explicit limits for intron or exon definition [76,77].

### TE clusters as exonization targets

TEs have been exapted as coding and regulatory sequences in many host genes [16]. For example, LTRs were detected in ~250 exons of human protein-coding genes [15,60,61] and can also act as transcriptional promoters and enhancers, often in a tissue-specific manner [78]. A number of exons of long non-coding RNAs originated from LTRs [79]. The exonized LTRs employ diverse sets of splice sites and their exonization levels are relatively high, yet significantly lower than those exhibited by *Alu*s [15].

We have shown that disease-causing exonizations are not driven only by a single TE or a single TE family. The birth of new *F8* exon was contingent on the presence of both LTR78 and *Alu*J copies (Figure 3(a,b)). LTR78 has ~5,000 copies in the human genome and has been found in many mammals [80] whereas the *Alu*J exonization partner is more abundant and younger [15]. As the most ancient *Alu* subfamily, *Alu*J elements are overrepresented among *Alu* exons [15,17,18]. By aligning primate *F8* intron 18 orthologs, we found that the *Alu*J copy was absent in tarsier, mouse lemur and bushbaby genomes, indicating that the transposition took place before the split of New and Old World Monkeys just over 40 million years ago. The same evolutionary period was implicated in the exonization of other alternatively spliced *Alu*s, such as in the *RPE* gene [81]. Thus, the time lag between LTR78 and *Alu*J insertions might even exceed 100 million years of animal evolution. LTRs are underrepresented in introns relative to other TEs and intronic LTRs are predominantly in the antisense orientation [15,82], as in the observed case (Figure 3(a,b)). In contrast, sense LTR78 sequences were reported to be overrepresented in exons of long non-coding RNAs [79]. Interestingly, the co-option of LTR78 in transcription regulatory sequences was associated with their tissue-specific expression [80]. Besides the mutually dependent adoption of LTR and *Alu* copies into the new exon (Figure 3), the two TEs can be exapted independently, as exemplified by neuronal-specific enhancers that dictate *Pomc* expression in the hypothalamus of placental mammals, with a time lag between evolution of the two enhancer modules estimated at ~20 million years [83].

The exonized TE cluster shown in Figure 3(c,d) is the first example of an exonized MER58A element. The human genome contains ~12,000 MER58A fragments, but only a hundred of them interrupt an older TE [84]. Finally, updated DBASS data illustrate that mutations activating pseudoexons derived from TEs and their clusters can be found either within or outside TEs and that in addition to supplying full splicing recognition motifs, TEs can contribute only their portions, enhancing combinatorial diversity and functional potential of new coding sequences (Figure 4(a-d)).

### Activating new 5’ss by destabilizing the 7SL RNA progeny

The WT counterpart of the LTR78/*Alu*J pseudoexon 5’ss (AAA/GCAAGA) is not used *in vivo*, as excluded by RT-PCR [62]. Nevertheless, ~1% of human introns are spliced out via GC 5’ss [85]. Their efficient removal requires more robust traditional and auxiliary splicing motifs nearby that compensate for their reduced instrinsic strength [85,86]. Mutation C > T at intron position +2 is the most important alteration required for exonization of both antisense and sense *Alu*s via new 5’ss [87]. It corrects the central mismatch between the U1 small nuclear RNA and the 5’ss consensus, which improves stability of the duplex more than those further away from the centre [69 and references therein]. Our SHAPE-guided predictions suggest that while most positions of the GC 5’ss motif in the WT *F8* are base-paired in the conserved 7SL RNA-derived stem (Figure 7(a,b)), the mutated site is more accessible (Figures 6, 7(c), and S1). The rearrangement should therefore improve the U1:5’ss base-pairing and potentially stabilize interactions of other U1 components, including those that may not make direct contacts with pre-mRNA bases but further enhance 5’ss affinities, such as U1-C [69].

The *Alu* domain of SRP RNA moiety is conserved in eukaryotes and archaea, but not in all eubacteria [66]. Bacterial and archaeal genomes were invaded by group II introns, almost assuredly ancestors of mammalian spliceosomes and nuclear introns [88,89]. This invasion was a defining event in the evolution of eukaryotes and alternative splicing [88,89]. Assuming the estimated evolutionary age of archaea [90], the C > U substitution in the primate-specific 7SL RNA progeny was thus sufficient for the high-inclusion exonization of a ~ 2 billion years-old RNA helix (Figures 3(a,b) and 7). This helix is an integral part of the core RNA structure of the *Alu* domain that maintained the highly conserved fold to date. Our comparison with published structures of the *Alu* domain RNAs [68,91] revealed that the decoy 5’ss homologous to *F8* was present already in archaeon *M. jannaschii* (CAU/GCCCAC). The paired configuration of the primordial 5’ss consensus can even be traced back to some eubacteria, such as *B. subtillis*, where the *Alu* domain is stabilized by prokaryote-specific 7SL RNA extensions that make interacting proteins dispensable [68,91].

Our comparison of *F8 Alu*J with 7SL RNA and existing *Alu* exons suggests that release of decoy 5’ss from H3.3 took place not only in *Alu*J exons, but also in free left *Alu* monomers and younger subfamilies (*Alu*S and *Alu*Y; Table S2, Fig. S3 and Figure 7(d)). For example, an *Alu*S copy in alternatively spliced *PKP2* transcripts was co-opted after the split of *Cercopithecoidea* and *Hominoidea* [81]. The 3’ss of this exon was contributed by an intronic sequence not recognized as a TE [81], unlike the composite exon in *F8*, further suggesting that high exon inclusion levels observed for the LTR78/*Alu*J exon [62] reflect the presence of a polypyrimidine-rich portion of LTR78. A lack of correlation between ME scores of 5’ss of exonized *Alu*s and their inclusion levels (r = −0.09, Table S2) also points to the importance of their 3’ss and cross-exon motifs. Finally, apart from the left arms of sense *Alu*, a homologous decoy 5’ss was likely used for exonization of their right arm, as exemplified by *Alu*S in *TBL1Y* (ref. [60]), although the underlying structural rearrangement needs confirmation.

Our structural probing suggested that the H3.3 ortholog in *F8 Alu*J could not sustain a swap of the central GC base pair for the wobble GU pair (Figures 6–7) although we cannot exclude that the stem is maintained in a subpopulation of RNAs. Thermodynamic stabilities of GU base pairs are lower than GC base pairs, however, GU pairs have a greater potential for RNA–RNA and RNA–protein interactions as a result of their higher structural flexibility and unique electrostatic landscape and geometry, manifested as nonisostericity and local over- or underwinding [92,93]. GU pairs do not always form base pairs using their Watson-Crick edges, particularly if surrounded by a single-stranded region, and their function is better compensated by AU pairs than by less flexible GC pairs [94]. Nearly all GU wobble pairs in a 359-nt viroid RNA were critical for replication or systemic spread [94], highlighting their functional importance. Conserved GU pairs identify cleavage sites of self-splicing introns and bind metal ions; metal ion catalysis is common in large ribozymes [95–98].

The *Alu* domain is responsible for the elongation arrest activity of SRP by interfering with elongation factor binding to the ribosome. In the hierarchical assembly model, the 3ʹ part of the mammalian *Alu* domain (including H3.3 or H5b) flips back onto the highly flexible 5ʹ portion upon binding of the SRP9/14 heterodimer [99]. The heterodimer stabilizes the *Alu* domain fold [99] and probably associates with all cytoplasmic *Alu*-like RNAs, including BC200 [66]. It first binds a three-way RNA junction connected by a central U-turn (Figure 7(a)), inducing or stabilizing H2/H1 stacking interactions [99,100]. In the subsequent assembly step, the 3ʹ part of the *Alu* domain folds back up to 180 degrees to contact the 5ʹ portion and SRP9, progressing into the closed *Alu* RNP conformation [99]. Interestingly, H3.3 contains RNase V1 cleavage sites protected in the presence of SRP9/14 but only if the link to the *Alu* RNA 5ʹ domain remains flexible [99].

The left and right arms of *Alu* elements appear to fold independently, each maintaining the overall cruciform 7SL RNA structure [65]. However, *Alu* dimers seem to provide a much more efficient substrate for splice-site activation than expected for *Alu* monomers alone, arguing for a thus far unexplained synergistic effect [15]. The antisense right arms contributed most *Alu* exons; they were activated more frequently through 3’ss with PPTs derived from antisense polyA tails [15], rather than via 5’ss. Sense *Alu*s do not enjoy a luxury of PPT-driven support of antisense copies [15]; to exonize, they need to get this help from elsewhere, such as anonymous intronic sequences [81] or LTR (Figure 3). As the 5ʹ linker appeared to pair with native RNA (Figure 7(b,c)), a reliable structural information for the 5ʹ exon portion and the 3’ss could not be obtained, which will need further studies. Nevertheless, the predicted GAGG tetraloop in both *F8* probes is supported by stable stem(s) consisting of 7 contiguous base pairs (Figure 7(b,c)). Such long helices are required for ultrarapid annealing in both RNA and DNA [101], suggesting that the hairpin is likely to form *in vivo*, at least for a limited time after transcription. In the SRP RNA, however, the GAGG motif is a part of the 3-way RNA junction (Figure 7(a-c)). Speculatively, the absence of homologous RNA junction in the *F8 Alu*J copy would open up the structure, potentially precluding SRP9/14 binding and formation of the closed conformation. Future studies should also characterize interactions affected by the C > U mutation in H3.3 orthologs in more detail, both with protein and RNA *trans*-acting factors, and address how exactly the 5’ss and auxilliary motifs upstream cooperate when no longer held together by base-pairing.

In conclusion, we report that a single-nucleotide substitution unleashed a decoy GC 5’ss motif that was hidden by Watson-Crick base-pairing in the central stem of 7SL RNA ~2 billion years before the 5’ss could become activated in the primate central stem progeny and cause haemophilia. The paired conformation of decoy GC 5’ss and its primordial exon repressor sequences in the opposite strand and 7SL RNA terminus can be traced back into secondary structures of archaea and eubacteria, *ie*. organisms that lack spliceosomal introns.

## Supplementary Material

Supplemental Material

## Data Availability

The datasets generated and/or analysed during the current study are in the Database of Aberrant Splice Sites (DBASS), which is freely available to view online at http://dbass.org.uk. The web site is mirrored at http://dbass.soton.ac.uk. Direct URLs for DBASS3 and DBASS5 are at http://dbass3.soton.ac.uk and http://dbass5.soton.ac.uk.

## Code availability

Code reported in this work is available in File S1.
