# Supplementary material for: Transposon clusters as substrates for aberrant splice-site activation
Source: RNA Biol. 2020 Sep 23;18(3):354–67. doi: 10.1080/15476286.2020.1805909 (PMC7951965; doi:10.1080/15476286.2020.1805909)
Supplement: Supplemental Material [file KRNB_A_1805909_SM0818.zip › Supplementary information/File S1.pdf]

run.sh

```
#!/bin/bash
#need a whole genome fasta to create local BLAST database
fasta='~/Documents/Work/Databases/GRCh38.primary_assembly.genome.fa'
#install ncbi command line blast
sudo apt-get install ncbi-blast+
git clone https://github.com/VCCRI/Spliceogen.git Spliceogen
#create database
makeblastdb -in ../../Databases/GRCh38.primary_assembly.genome.fa -dbtype nucl -out hg38whole
#make queryFile
inputFile="db3.txt"
rm "$inputFile"_temp1.txt
#remove empty lines and '(5'ss)', quotes etc
cat "$inputFile" | grep -v Sequence | sed '/^[[:space:]]*$/d' | sed -e "s/(3?ss1)//g" | sed -e
"s/(3?ss2)//g" | sed -e "s/(3?ss3)//g" | sed -e "s/(3'ss)//g" | sed -e "s/(3'SS)//g" | sed -e "s/(5?ss1)//g" |
sed -e "s/(5?ss2)//g" | sed -e "s/(5?ss3)//g" | sed -e "s/(5'ss)//g" | sed -e "s/(5'SS)//g" | tr -d '"' >
"$inputFile"_temp1.txt
prevLine=""
rm "$inputFile"_temp2.txt
head -1 "$inputFile" > "$inputFile"_temp2.txt
#merge sequences split over multiple lines onto one line
re='^[0-9]+$'
while read -r line; do
    #check if current line starts with a number
    checkNewline=$(echo "$line" | awk '{print $1}')
    if [[ $checkNewline =~ $re ]] ; then
        echo "$prevLine" >> "$inputFile"_temp2.txt
        prevLine="$line"
    else
        prevLine=$(echo "$prevLine" "$line" | tr -d '\n')
    fi
done<"$inputFile"_temp1.txt
#print final line
echo "$prevLine" >> "$inputFile"_temp2.txt
#handle gapped inputs
grep -v '\.' "$inputFile"_temp2.txt > "$inputFile"_ungapped.txt
grep '\.' "$inputFile"_temp2.txt > "$inputFile"_gapped.txt
#inputs with large gaps were manually split/edited here.
cat "$inputFile"_gappedInputs_manuallyEdited.txt >> "$inputFile"_ungapped.txt
rm query.txt
while read -r line; do
    #ignore header
    headerIgnore=$( echo "$line" | grep Sequence)
    if [ "$headerIgnore" == "" ]; then
        fastaId=$(echo "$line" | awk -F'\t' '{print $1}')
        fastaSeq=$(echo "$line" | awk -F'\t' '{print $3}')
        echo '>'$fastaId >> query.txt
        echo "$fastaSeq" >> query.txt
    fi
done<"$inputFile"_ungapped.txt
python cleanSeqForBlast.py query.txt > query_cleaned.txt
```

```
echo "running Blastn"
time blastn -db ~/Documents/Work/dbass/Blast_Tutorial/hg38whole -query query_cleaned.txt -
max_target_seqs 1 -max_hsps 1 -outfmt 6 | sort -k1,1 > "$inputFile"_blast.out
echo "finished running Blastn"
rm "$inputFile"_temp.txt
while read -r line; do
    id=$(echo $line | awk '{print $1}')
    chr=$(echo $line | awk '{print $2}')
    fastaStart=$(echo $line | awk '{print $9}')
    fastaEnd=$(echo $line | awk '{print $10}')
    gap=$(echo $line | awk '{print $6}')
    echo -e "$id\t$chr\t$fastaStart\t$fastaEnd\t$seq\t$gap" >> "$inputFile"_temp.txt
done<"$inputFile"_blast.out
echo -e "#id\tchr\tref\talt\tn1\tn2\tn3\tn4\tn5\ttestVar\ttestSite" > "$inputFile"_align.out
python align.py "$inputFile"_temp.txt "$inputFile"_temp2.txt  >> "$inputFile"_align.out
#output variant positions
#snvs
cat "$inputFile"_align.out | grep -v ref | awk -v OFS="\\t" '{print $2, $7, $3, $4}' | grep -v ',' | grep -
v '\.' | sort -u > Spliceogen/"$inputFile"_out.tsv
#indels
cat "$inputFile"_align.out | grep -v ref | awk -v OFS="\\t" '{print $2, $8, $3, $4}' | grep -v ',' | grep -
v '\.' | sort -u >> Spliceogen/"$inputFile"_out.tsv
#TESTING... using the sequence adjacent to n1/n3/n4, create a deletion at the variant position to be
tested using Spliceogen's reference allele check
#snvs
cat "$inputFile"_align.out | grep -v ref | awk -v OFS="\\t" '{print $2, $7, $10, $3}' | grep -v ',' | grep
-v '\.' | sort -u > Spliceogen/"$inputFile"_variantTest.tsv
#deletions
cat "$inputFile"_align.out | grep -v ref | awk 'length($4) == 1 { print }' | awk -v OFS="\\t" '{print
$2, $8, $10, $4}' | grep -v ',' | grep -v '\.' | sort -u >> Spliceogen/"$inputFile"_variantTest.tsv
#insertions
cat "$inputFile"_align.out | grep -v ref | awk 'length($4) >= 2 { print }' | awk -v OFS="\\t" '{print
$2, $8, $10, $4}' | grep -v ',' | grep -v '\.' | sort -u >> Spliceogen/"$inputFile"_variantTest.tsv
#n1
cat "$inputFile"_align.out | grep -v ref | awk -v OFS="\\t" '{print $2, $6, $11, substr($11,1,2)}' |
grep -v ',' | grep -v '\.' | sort -u > Spliceogen/"$inputFile"_n1Test.tsv
#output in ordered excel format
rm "$inputFile"_orderedOut.txt
rm Spliceogen/"$inputFile"_n1Test.tsv
rm Spliceogen/"$inputFile"_variantTest_multiple.tsv
while read -r id; do
    match=$(grep -E "^$id[[:space:]]" "$inputFile"_align.out | head -1)
    #echo "id, match: $id $match" >> 5.out
    chr=$(echo "$match" | awk '{print $2}')
    n1=$(echo "$match" | awk '{print $5}')
    n2=$(echo "$match" | awk '{print $6}')
    n3=$(echo "$match" | awk '{print $7}')
    n4=$(echo "$match" | awk '{print $8}')
    n5=$(echo "$match" | awk '{print $9}')
    ref=$(echo "$match" | awk '{print $3}')
    alt=$(echo "$match" | awk '{print $4}')
    site=$(echo "$match" | awk '{print $11}')
```

```
    varTest=$(echo "$match" | awk '{print $10}')
    pos=$(echo "$n3")
    if [ "$pos" == "." ]; then
        pos=$(echo "$n4")
    fi
    #handle multiple vars/sites
    n1arr=(${n1//,/ })
    n2arr=(${n2//,/ })
    n3arr=(${n3//,/ })
    refArr=(${ref//,/ })
    altArr=(${alt//,/ })
    siteArr=(${site//,/ })
    varTestArr=(${varTest//,/ })
    n1out=""
    refAlt=""
    #format ref>alt
    for i in $(seq 1 ${#refArr[@]}); do
        if [ "$refAlt" != "" ]; then
            refAlt="$refAlt,"
        fi
        let "j = $i - 1"
        refAlt="$refAlt${refArr[$j]}>${altArr[$j]}"
        if [ "${#n3arr[@]}" -gt "1" ]; then
            echo -e "$chr\t${n3arr[$j]}\t${varTestArr[$j]}\t${refArr[$j]}" >>
Spliceogen/"$inputFile"_variantTest_multiple.tsv
        fi
    done
    #format n1/n2
    for i in $(seq 1 ${#n1arr[@]}); do
        if [ "$n1out" != "" ]; then
            n1out="$n1out,"
        fi
        let "j = $i - 1"
        n1out="$n1out${n1arr[$j]}/${n2arr[$j]}"
        echo -e "$chr\t${n2arr[$j]}\t${siteArr[$j]}\tA" >> Spliceogen/"$inputFile"_n1Test.tsv
    done
    echo -e "$id\t$chr:$pos\t$n1out\t$n1\t$n2\t$refAlt\t$n3\t$n4\t$n5" | tr -d '.' >>
"$inputFile"_orderedOut.txt
done<"$inputFile"_id.txt
cd Spliceogen
gtf="../../Databases/gencode.v30.basic.annotation.gtf.gz"
#run out. and try +/-1 offsets
./RUN.sh -input "$inputFile"_out.tsv -gtf "$gtf" -fasta "$fasta"
#for ref mismatches, try a plus one offset
cat output/"$inputFile"_out.tsvrefMismatch.txt | sed 's/>//g' | tr ';' '\t' | awk -v OFS="\\t" '{print $1,
$2+1, $3, $4}' > "$inputFile"_out_plusOne.tsv
#run plus one offset
./RUN.sh -input "$inputFile"_out_plusOne.tsv -gtf "$gtf" -fasta "$fasta"
#for ref mismatches, try a minus one offset
cat output/"$inputFile"_out_plusOne.tsvrefMismatch.txt | sed 's/>//g' | tr ';' '\t' | awk -v OFS="\\t"
'{print $1, $2-2, $3, $4}' > "$inputFile"_out_minusOne.tsv
#run minus one offset
```

```
./RUN.sh -input "$inputFile"_out_minusOne.tsv -gtf "$gtf" -fasta "$fasta"
#run variant test. and try +/-1 offsets
./RUN.sh -input "$inputFile"_variantTest.tsv -gtf "$gtf" -fasta "$fasta"
#for ref mismatches, try a plus one offset
cat output/"$inputFile"_variantTest.tsvrefMismatch.txt | sed 's/>//g' | tr ';' '\t' | awk -v OFS="\\t"
'{print $1, $2+1, $3, $4}' > "$inputFile"_variantTest_plusOne.tsv
#run plus one offset
./RUN.sh -input "$inputFile"_variantTest_plusOne.tsv -gtf "$gtf" -fasta "$fasta"
#for ref mismatches, try a minus one offset
cat output/"$inputFile"_variantTest_plusOne.tsvrefMismatch.txt | sed 's/>//g' | tr ';' '\t' | awk -v
OFS="\\t" '{print $1, $2-2, $3, $4}' > "$inputFile"_variantTest_minusOne.tsv
#run minus one offset
./RUN.sh -input "$inputFile"_variantTest_minusOne.tsv -gtf "$gtf" -fasta "$fasta"
#run n1 test
./RUN.sh -input "$inputFile"_n1Test.tsv -gtf "$gtf" -fasta "$fasta"
#for ref mismatches, try a plus one offset
cat output/"$inputFile"_n1Test.tsvrefMismatch.txt | sed 's/>//g' | tr ';' '\t' | awk -v OFS="\\t" '{print
$1, $2+1, $3, $4}' > "$inputFile"_n1Test_plusOne.tsv
#run plus one offset
./RUN.sh -input "$inputFile"_n1Test_plusOne.tsv -gtf "$gtf" -fasta "$fasta"
#for ref mismatches, try a minus one offset
cat output/"$inputFile"_n1Test_plusOne.tsvrefMismatch.txt | sed 's/>//g' | tr ';' '\t' | awk -v OFS="\\t"
'{print $1, $2-2, $3, $4}' > "$inputFile"_n1Test_minusOne.tsv
#run minus one offset
./RUN.sh -input "$inputFile"_n1Test_minusOne.tsv -gtf "$gtf" -fasta "$fasta"
```

align.py

```
#!/usr/bin/python
import sys, re
import pandas as pd
from Bio.Seq import Seq
inputFile = sys.argv[1]
seqIdFile = sys.argv[2]
f = open(inputFile, 'r')
line = f.readline()
#read seq and IDs
seqId = pd.read_csv(seqIdFile, sep="\t")
seqId.ID = seqId.ID.astype(str)
while (line):
    id = re.split(r'\t+', line)[0]
    chr = re.split(r'\t+', line)[1]
    seq = str(seqId.loc[seqId['ID'] == str(id), 'Sequence'].iloc[0].replace(" ","").replace("\n",""))
    seq = seq.strip('.')
    pos1 = int(re.split(r'\t+', line)[2])
    pos2 = int(re.split(r'\t+', line)[3])
    fastaStart = pos1
    fastaEnd = pos2
    reverseStrand = False
    if (pos1 > pos2):
        reverseStrand = True
        seq = str(Seq(seq).reverse_complement())
        fastaStart = pos2
        fastaEnd = pos1
    start = refOut = altOut = n1 = n2 = n3 = n4 = n5 = testVar = testSite = ''
    def getStartPos(s):
        #remove insertions and dashes from string, return position of final nt
        s = s.replace('/','')
        insertion = s.find('[')
        while insertion != -1:
            s = re.sub('\[[^\]]+\]', '', s, 1)
            insertion = s.find('[')
        pos = len(s) + fastaStart + 1
        #if reverseStrand:
        #    pos = pos - 1
        return pos
    if reverseStrand:
        #substitutions and deletions
        var = re.search(".[)].*?.[(]", seq)
        while var is not None:
            var = var.group(0)
            trailing = seq.split(var,1)[1]
            #deletions
            if '>' not in var:
                ref = var.split('(')[0].replace(')','').replace('(','')
                alt = var.split(')')[0]
                delStart = getStartPos(seq.split(var, 1)[0]) -1
                delEnd = len(ref) + delStart
```

```
            if not n4:
                n4 = str(delStart)
                n5 = str(delEnd)
            else:
                n4 = n4 + "," + str(delStart)
                n5 = n5 + "," + str(delEnd)
            #replace variant with ref allele in seq
            seq = seq.replace(var,ref,1)
        #substitutions
        else:
            alt = var.split('>')[0].split(')')[1]
            ref = var.split('>')[1].split('(')[0]
            startPos = getStartPos(seq.split(var, 1)[0])
            replaceStr = var.split(')')[0] + ref
            if '/' in replaceStr:
                startPos = startPos - 1
            if not n3:
                n3 = str(startPos)
            else:
                n3 = n3 + "," + str(startPos)
            #replace variant with ref allele in seq
            seq = seq.replace(var,replaceStr,1)
        #update ref/alt
        if not refOut:
            refOut = ref
            altOut = alt
        else:
            refOut = refOut + "," + ref
            altOut = altOut + "," + alt
        #check for another substitution/deletion
        var = re.search('.\).*?\(', seq)
        #test chars
        trailing = trailing[:5].replace("/","")
        if not testVar:
            testVar = ref + trailing
        else:
            testVar = testVar + "," + ref + trailing
    #cryptic/de novo sites
    site = seq.find('/')
    while site != -1:
        n1pos = int(fastaStart) + site - 1
        n2pos = int(fastaStart) + site
        siteSeq = seq[site+1:site+6]
        if not n1:
            n1 = str(n1pos)
            n2 = str(n2pos)
        else:
            n1 += "," + str(n1pos)
            n2 += "," + str(n2pos)
        if not testSite:
            testSite = siteSeq
        else:
```

```
            testSite = testSite + "," + siteSeq
        seq = seq.replace('/',"",1)
        site = seq.find('/')
    #insertions
    insert = re.search('.\].*?\[', seq.strip('\/'))
    while insert is not None:
        insert = insert.group(0)
        ref = insert.split(']')[0]
        alt = ref + insert.split(']')[1].split('[')[0]
        startPos = getStartPos(seq.split(insert, 1)[0]) - 1
        endPos = startPos + len(alt)
        if not n4:
            n4 = str(startPos)
            n5 = str(endPos)
        else:
            n4 = n4 + "," + str(startPos)
            n5 = n5 + "," + str(endPos)
        #replace variant with ref in fasta
        seq = seq.replace(insert,'',1)
        #check for another variant
        insert = re.search('.\].*?\[', seq)
        #update ref/alt
        if not refOut:
            refOut = ref
            altOut = alt
        else:
            refOut = refOut + "," + ref
            altOut = altOut + "," + alt
    refOut = refOut.replace('/','')
    altOut = altOut.replace('/','')
    out = [n1, n2, n3, n4, n5, testVar, testSite]
    for i in range(0,7):
        if not out[i]:
            out[i] = "."
    print(id, chr, refOut, altOut, out[0], out[1], out[2], out[3], out[4], out[5], out[6], sep='\t')
    line = f.readline()
#sense strand
else:
    #substitutions and deletions
    var = re.search('.\(.*?.\)', seq)
    while var is not None:
        var = var.group(0)
        trailing = seq.split(var,1)[1]
        trailing = trailing[:5].replace("/","")
        #deletions
        if '>' not in var:
            ref = var.split(')')[0].replace('(','').replace(')','')
            alt = var.split('(')[0]
            delStart = getStartPos(seq.split(var, 1)[0])
            delEnd = len(ref) + delStart -1
            if not n4:
                n4 = str(delStart)
```

```
            n5 = str(delEnd)
        else:
            n4 = n4 + "," + str(delStart)
            n5 = n5 + "," + str(delEnd)
        #replace variant with ref allele in seq
        seq = seq.replace(var,ref,1)
    #substitutions
    else:
        ref = var.split('>')[0].split('(')[1]
        alt = var.split('>')[1].split(')')[0]
        startPos = getStartPos(seq.split(var, 1)[0])
        replaceStr = var.split('(')[0] + ref
        if '/' in replaceStr:
            startPos = startPos - 1
        if not n3:
            n3 = str(startPos)
        else:
            n3 = n3 + "," + str(startPos)
        #replace variant with ref allele in seq
        seq = seq.replace(var,replaceStr,1)
    #update ref/alt
    if not refOut:
        refOut = ref
        altOut = alt
    else:
        refOut = refOut + "," + ref
        altOut = altOut + "," + alt
    #check for another substitution/deletion
    var = re.search('.\(.*?\)', seq)
#cryptic/de novo sites
site = seq.find('/')
while site != -1:
    n1pos = int(fastaStart) + site - 1
    n2pos = int(fastaStart) + site
    siteSeq = seq[site+1:site+6]
    if not n1:
        n1 = str(n1pos)
        n2 = str(n2pos)
    else:
        n1 += "," + str(n1pos)
        n2 += "," + str(n2pos)
    if not testSite:
        testSite = siteSeq
    else:
        testSite = testSite + "," + siteSeq
    seq = seq.replace('/',"",1)
    site = seq.find('/')
#insertions
insert = re.search('.\[.*?\]', seq.strip('\/'))
while insert is not None:
    insert = insert.group(0)
    ref = insert.split('[')[0]
```

```
            alt = ref + insert.split('[')[1].split(']')[0]
            startPos = getStartPos(seq.split(insert, 1)[0])
            endPos = startPos + len(alt)
            if not n4:
                n4 = str(startPos)
                n5 = str(endPos)
            else:
                n4 = n4 + "," + str(startPos)
                n5 = n5 + "," + str(endPos)
            #replace variant with ref in fasta
            seq = seq.replace(insert,ref,1)
            #update ref/alt
            if not refOut:
                refOut = ref
                altOut = alt
            else:
                refOut = refOut + "," + ref
                altOut = altOut + "," + alt
            #check for another variant
            insert = re.search('.\[.*?\]', seq)
        refOut = refOut.replace('/','')
        altOut = altOut.replace('/','')
        #need to test for / within variant
        if not testVar:
            testVar = ref + trailing
        else:
            testVar = testVar + "," + ref + trailing
        out = [n1, n2, n3, n4, n5, testVar, testSite]
        for i in range(0,7):
            if not out[i]:
                out[i] = "."
        print(id, chr, refOut, altOut, out[0], out[1], out[2], out[3], out[4], out[5], out[6], sep='\t')
        line = f.readline()
f.close()
```

```
#!/usr/bin/python
import sys, re
import pandas as pd
inputFile = sys.argv[1]
f = open(inputFile, 'r')
line = f.readline()
while (line):
    if line.startswith('>'):
        print(line.rstrip())
        line = f.readline()
    else:
        #substitutions and deletions
        #remove leading and trailing ellipses
        line = line.strip('.')
        var = re.search('\(.*?.\)', line)
        while var is not None:
            var = var.group(0)
            #deletions
            if '>' not in var:
                ref = var.split(')')[0].replace('(',").replace(')',")
            #substitutions
            else:
                ref = var.split('>')[0].split('(')[1]
            #replace variant with ref allele in seq
            line = line.replace(var,ref,1)
            #check for another substitution/deletion
            var = re.search('\(.*?\)', line)
        #cryptic/de novo sites
        site = line.find('/')
        while site != -1:
            line = line.replace('/',",1)
            site = line.find('/')
        #insertions
        insert = re.search('\[.*?\]', line.strip('\/'))
        while insert is not None:
            insert = insert.group(0)
            ref = insert.split('[')[0]
            #replace variant with ref in fasta
            line = line.replace(insert,ref,1)
            #check for another insertion
            insert = re.search('\[.*?\]', line)
        print(line.rstrip().replace(" ",""))
        line = f.readline()
f.close()
```
