# Supplementary material for: Transposon clusters as substrates for aberrant splice-site activation
Source: RNA Biol. 2020 Sep 23;18(3):354–67. doi: 10.1080/15476286.2020.1805909 (PMC7951965; doi:10.1080/15476286.2020.1805909)
Supplement: Supplemental Material [file KRNB_A_1805909_SM0818.zip › Supplementary information/Supplemental Figures 1-3.pdf]

Transposon clusters as substrates for mutation-induced aberrant splice-site activation

SUPPLEMENTARY FIGURES

Maria Elena Vilar Alvarez, Martin Chivers, Ivana Borovska, Steven Monger, Eleni Giannoulatou, Jana Kralovicova and Igor Vorechovsky

Supplementary Fig. S1 Structural probing of WT and mutated LTR78/*Alu*J RNAs with RNase A

a Polyacrylamide gel electrophoresis with WT and mutated (C>U) *F8* products (mock)-digested with RNase A (10x dilutions for each probe). The products are numbered to the right. The same numbers denote cleavage sites in the secondary structure models in Fig. 7b,c. **b** The ratios of mean signal intensities of the indicated products in the mutant *vs.* WT. Mean signals between cleavage sites were significantly different (P<0.0001, ANOVA with Tukey's post-hoc tests). Dotted line represents equal signals from the mutant and the WT.

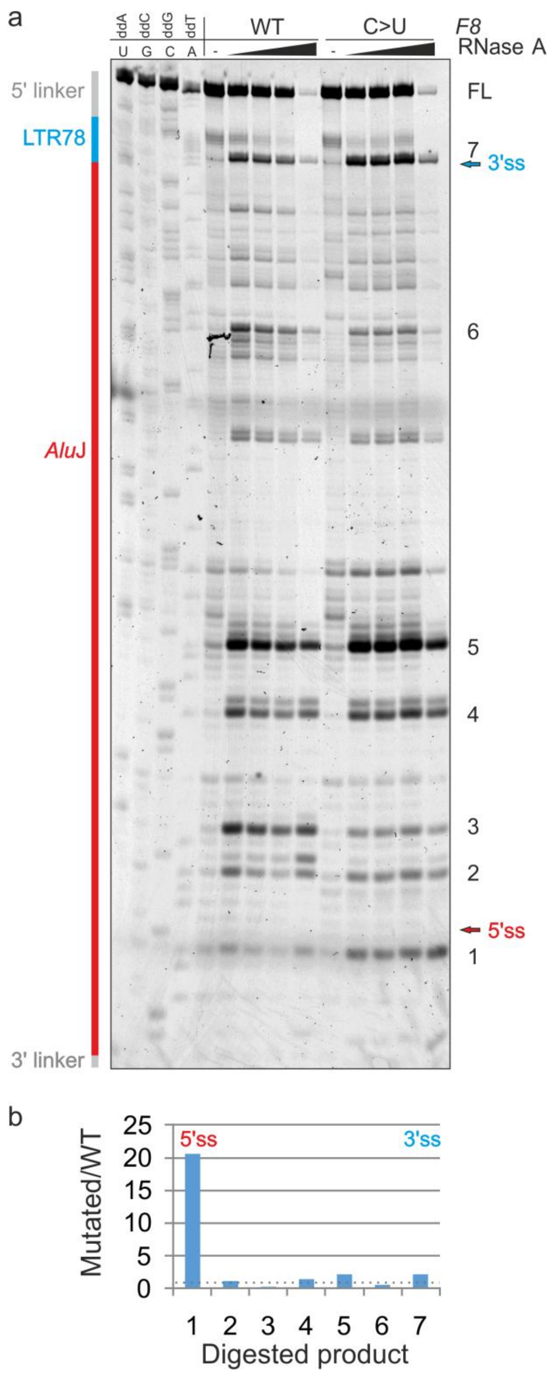

Supplementary Fig. S2 Alternative SHAPE-guided secondary structures of the *F8* pseudoexon

a WT, **b** C>U mutant. The most stable SHAPE-guided structures are in Fig. 7b,c. Secondary structures were predicted by RNAstructure [1] using normalized NAI constraints and default options. The 5' and 3' linkers are highlighted in grey. Pseudoexon splice sites are shown as open green arrowheads; closed arrowheads in the WT denote their decoy counterparts. Black triangles indicate major RNase A cleavage sites (numbered 1-7 in Fig. S1); their size reflects normalized cleavage intensities in the WT. In the mutant (**b**), the triangle size indicates their changes from the WT; red triangles indicate >3-fold differences between the WT and mutant.

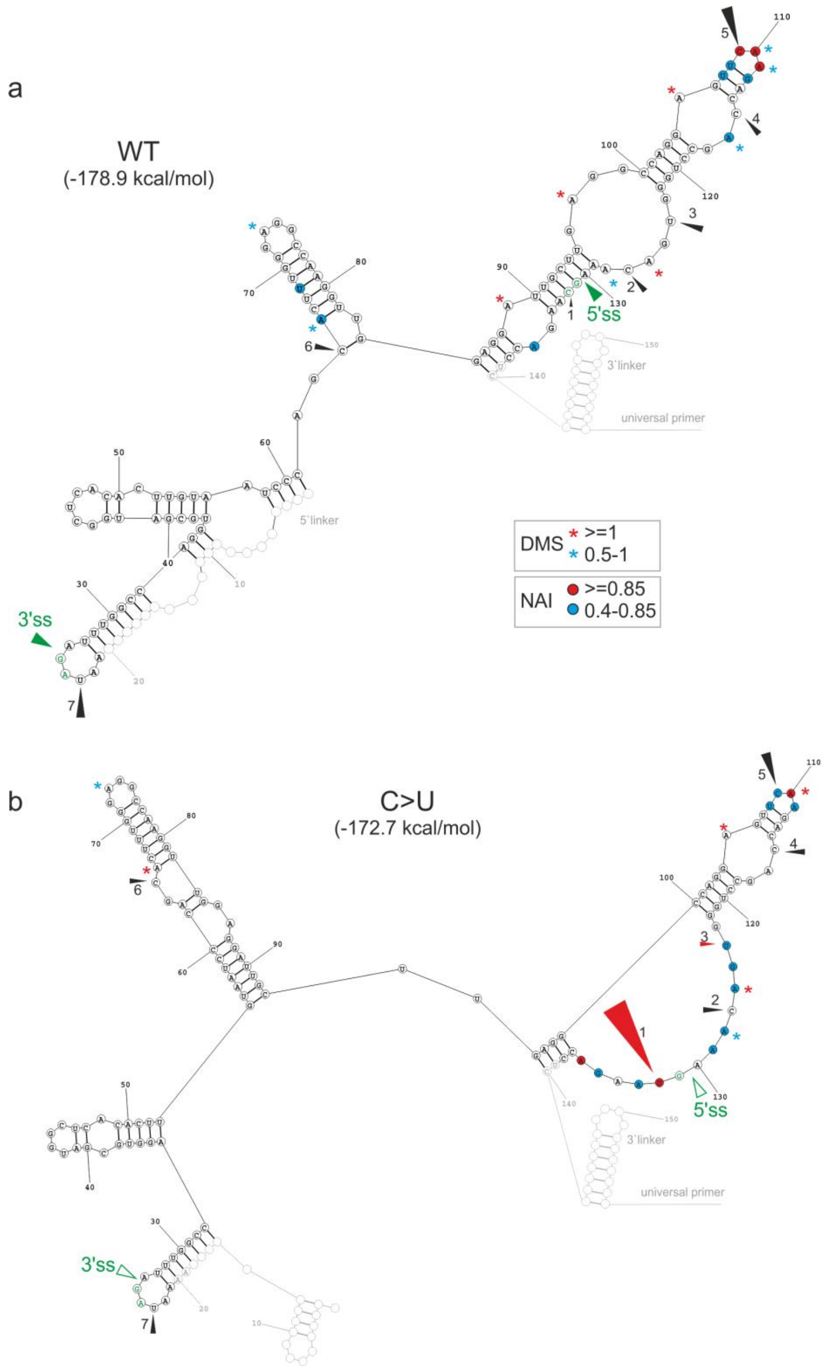

Supplementary Fig. S3 Multiple alignment of existing sense *Alu* exons that use 5’ss homolgous to the *F8* pseudoexon

Invariable intron position +2 is highlighted. *F8* triloop that borders the Alu-domain and S-domain in the RNA moiety of SRP is boxed. Exon inclusion values are in Table S2, except for *PKP2*. Alternative *Alu*S exon in *PKP2* encodes the end of an armadillo repeat of isoform B but not isoform A [2]. Evolution of this exon was described by Krull and co-workers [3].

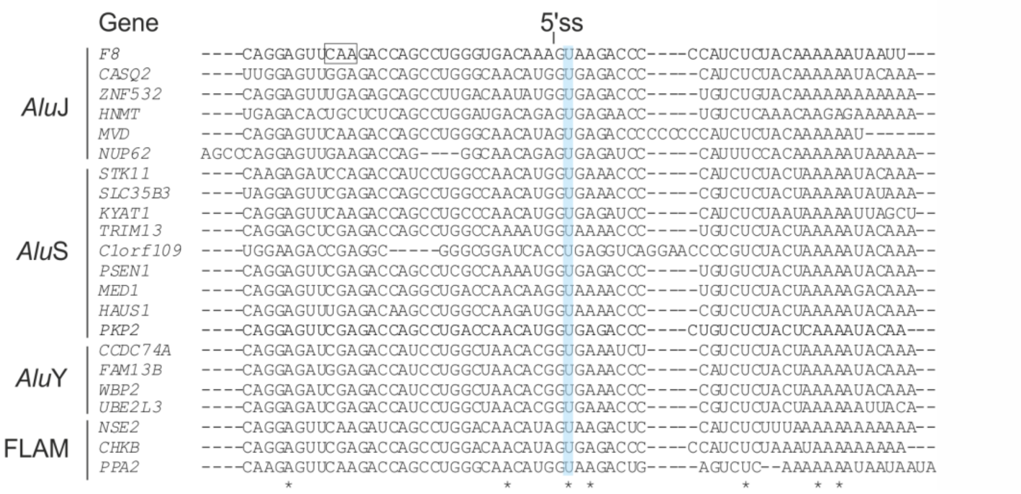

References to Supplementary figures

1. Mathews DH. RNA secondary structure analysis using RNAstructure. Curr Protoc Bioinformatics 2006; 12:12.6.

2. Mertens C, Kuhn C, Franke WW. Plakophilins 2a and 2b: constitutive proteins of dual location in the karyoplasm and the desmosomal plaque. J Cell Biol 1996; 135:1009-25.

3. Krull M, Brosius J, Schmitz J. Alu-SINE exonization: en route to protein-coding function. Mol Biol Evol 2005; 22:1702-11.
