# Supplementary material for: Transposon clusters as substrates for aberrant splice-site activation
Source: RNA Biol. 2020 Sep 23;18(3):354–67. doi: 10.1080/15476286.2020.1805909 (PMC7951965; doi:10.1080/15476286.2020.1805909)
Supplement: Supplemental Material [file KRNB_A_1805909_SM0818.zip › Supplementary information/Supplementary Table S1.pdf]

Gene symbol (synonyms)	Phenotype	Mutation	Aberrant splice site	ReadingFrameChange	delta MEauth and MEcr	Ensembl Gene Number	OMIM Number
ATP7B	Wilson's disease	c.3244-2A>G	3'ss	1	-8.59	ENSG00000123191	277900
BRCA2	Breast cancer	c.8954-3C>G	3'ss	2	-18.11	ENSG00000139618	600185
TGFB2	Loeys-Dietz syndrome type 4	c.839-1G>A	3'ss	1	-3.07	ENSG00000092969	614816
CYP21A2	Congenital adrenal hyperplasia	c.652-2A>G	3'ss	2	-8.96	ENSG00000231852	201910
GBA	Gaucher disease	c.1389-1G>A	3'ss	1	-2.96	ENSG00000177628	230800
DUOX2	Congenital hypothyroidism	c.2335-1G>C	3'ss	2	-0.13	ENSG00000140279	188450
AR	Androgen insensitivity syndrome	c.1769-1G>C	3'ss	0	-1.47	ENSG00000169083	300068
ABCC6	Pseudoexanthoma elasticum	c.1177-2A>G	3'ss	2	-27.13	ENSG00000091262	264800
ATP7B	Wilson disease	IVS11-2A>G	3'ss	0	-0.63	ENSG00000123191	277900
F12	Factor 12 deficiency	IVS13-1G>A	3'ss	1	-0.07	ENSG00000131187	234000
NTRK1 (TRKA)	Congenital insensitivity to pain with anhidrosis	IVS4-1G>C	3'ss	2	-1.42	ENSG00000198400	256800
TNFSF5 (HIGM1, CD40LG)	X-linked hyper-IgM syndrome	IVS4-2A>G	3'ss	2	-11.05	ENSG00000102245	308230
TSC2	Tuberous sclerosis	IVS9-3C>G	3'ss	2	-0.37	ENSG00000103197	191100
NF1	Neurofibromatosis, type I	IVS26-2A>T	3'ss	2	-0.57	ENSG00000196712	162200
RB1	Retinoblastoma	IVS12-3A>G	3'ss	2	-4.51	ENSG00000139687	180200
IVD	Isovaleric acidemia	IVS7-1G>A	3'ss	0	-0.35	ENSG00000128928	243500
DMD	Muscular dystrophy	IVS74-2A>G	3'ss	0	-3.2	ENSG00000198947	156225
RUNX1	Familial thrombocytopenia	IVS3-1G>T	3'ss	1	-1.69	ENSG00000159216	151385
SLC7A7	Lysinuric protein intolerance	IVS6-2A>T	3'ss	1	-1.51	ENSG00000155465	222700
IDS	Mucopolysaccharidosis, type II	IVS3-2A>G	3'ss	1	-3.4	ENSG00000010404	309900
HPRT1	Hypoxanthine-guanine phosphoribosyltransferase deficiency	IVS9-3T>G	3'ss	2	-0.34	ENSG00000165704	300322
SPG4 (SPAST)	Spastic paraplegia	IVS6-1G>A	3'ss	2	-1.09	ENSG00000021574	182601
MAN2B1	Alpha-mannosidosis	IVS14-2A>G	3'ss	1	-0.38	ENSG00000104774	248500
PKP2	Arrhytmogenic right ventricular cardiomyopathy	IVS12-1G>C	3'ss	1	-0.07	ENSG00000057294	602861
GLB1	Gangliosidosis	IVS14-2A>G	3'ss	1	-1.52	ENSG00000170266	230500
GCK	Maturity-onset diabetes of the young	IVS5-1G>A	3'ss	0	-0.02	ENSG00000106633	125853
TCIRG1	Osteopetrosis	IVS14-1G>A	3'ss	0	-1.45	ENSG00000110719	259700
FACL4	X-linked mental retardation	IVS10-2A>G	3'ss	1	-1.88	ENSG00000068366	300157
CCM2	Cerebral cavernous malformations	c.205-1_2delinsT	3'ss	1	-2.62	ENSG00000136280	603284
HRPT2 (CDC73)	Hyper-parathyroidism	IVS6-1delG	3'ss	2	-2.08	ENSG00000134371	145000
RB1	Retinoblastoma	g.64328A>G (IVS9-2A>G)	3'ss	1	-0.87	ENSG00000139687	180200
VWF	Von Willebrand disease	c.7082-2A>G	3'ss	1	-2.22	ENSG00000110799	613160
VWF	Von Willebrand disease	c.7730-1G>C	3'ss	2	-0.32	ENSG00000110799	277480
NF2	Neurofibromatosis type II	IVS11-2A>G	3'ss	1	-2.94	ENSG00000186575	101000
DMD	Muscular dystrophy	IVS32+1G>A	5'ss	2	-0.99	ENSG00000198947	181350
WISP3 (CCN6)	Progressive pseudorheumatoid dysplasia	E3+243G>C	5'ss	0	-0.71	ENSG00000112761	208230
GH-V (GH2)	Splice-site mutation in the human growth hormone-variant gene	IVS2+1G>A	5'ss	0	-1.75	ENSG00000136487	139240
ATP7A	Menkes disease	IVS6+5G>A	5'ss	2	-0.62	ENSG00000165240	309400
ADAMTS13	Thrombotic thrombocytopenic purpura	IVS13+5G>A	5'ss	0	-0.82	ENSG00000160323	274150
LPL	Lipoprotein lipase deficiency	IVS8+2T>C	5'ss	2	-2.4	ENSG00000175445	246650
IGHG2	Acute lymphoblastic leukemia	IVS1+4A>G	5'ss	1	-6.42	ENSG00000211893	147110
PSEN1	Alzheimer disease	IVS4+1delG	5'ss	1	-0.12	ENSG00000080815	607822
HBA2	Alpha-thalassemia	E1+135C>T	5'ss	1	-1.69	ENSG00000188536	141750
IDS	Mucopolysaccharidosis, type II	IVS7+5G>C	5'ss	1	-0.84	ENSG00000010404	309900
LGMD2B (DYSF)	Limb girdle muscular dystrophy	IVS45+5G>A	5'ss	1	-5.19	ENSG00000135636	253601
ABCD1	X-linked adrenoleukodystrophy	E1+99G>A	5'ss	0	-0.85	ENSG00000101986	300100
BTNL2	Sarcoidosis	E5+348G>A	5'ss	1	-1.22	ENSG00000204290	181000
RPGR	Primary ciliary dyskinesia	631_IVS6+9del	5'ss	2	-1.01	ENSG00000156313	242650
SEDL (TRAPPC2)	Spondyloepiphyseal dysplasia tarda	IVS4+4T>C	5'ss	1	-21.56	ENSG00000196459	313400
FRAS1	Fraser syndrome	IVS53+1G>T	5'ss	1	-3.05	ENSG00000138759	219000
COL1A1	Osteogenesis imperfecta	IVS8+5G>A	5'ss	0	-1.64	ENSG00000108821	166210
HMBS	Acute intermittent porphyria	IVS7+2T>C	5'ss	0	-0.05	ENSG00000149397	176000
ITGA2B	Glanzmann thrombasthenia	IVS10+5G>T	5'ss	2	-0.27	ENSG00000005961	273800
COL7A1	Epidermolysis bullosa	IVS51+1G>A	5'ss	2	-4.71	ENSG00000114270	120120
LDLR	Familial hypercholesterolemia	IVS10+5G>A	5'ss	0	-0.86	ENSG00000130164	144010
ITGB4	Epidermolysis bullosa	IVS30+1G>A	5'ss	0	-2.28	ENSG00000132470	120120
WASP	Wiskott-Aldrich syndrome	IVS6+2T>G	5'ss	2	-0.49	ENSG00000015285	301000
COL11A2	Otospondylomegaepiphyseal dysplasia	IVS53+5G>A	5'ss	2	-1.06	ENSG00000206290	215150
RB1	Retinoblastoma	del(E15-16_IVS15+37)	5'ss	0	-3.2	ENSG00000139687	180200
CYP19A1	Placental aromatase deficiency	IVS6+2T>C	5'ss	0	-0.98	ENSG00000137869	107910
DMD	Muscular dystrophy	IVS64+5G>C	5'ss	0	-2.37	ENSG00000198947	181350
ATM	Ataxia-telangiectasia	IVS28+159A>G	5'ss	2	-2.11	ENSG00000149311	208900
INS	Diabetes susceptibility	IVS1+5insTTGC	5'ss	0	-1.23	ENSG00000129965	125852
HEXA	Tay-Sachs disease	IVS9+1G>A	5'ss	2	-0.69	ENSG00000213614	272800
VHL	Von Hippel-Lindau disease	E2+122delA	5'ss	1	-1.32	ENSG00000134086	193300
HPRT1	Hypoxanthine phosphoribosyltransferase deficiency	IVS5+1delG	5'ss	0	-0.4	ENSG00000165704	300322
TCN2	Transcobalamin deficiency	IVS3+2T>G	5'ss	0	-0.51	ENSG00000185339	275350
BRCA1	Breast cancer predisposition	IVS7+1G>A	5'ss	2	-0.54	ENSG00000012048	113705
BRCA2	Breast cancer	IVS21+1G>A	5'ss	1	-1.02	ENSG00000139618	612555
SHANK3	Autism	IVS19+1delG	5'ss	1	-1.79	ENSG00000251322	209850
IDS	Mucopolysaccharidosis, type II	E3+178G>A	5'ss	2	-5.39	ENSG00000010404	309900
FTSJ1	X-linked mental retardation, nonsyndromic	IVS2+1delG	5'ss	1	-4.2	ENSG00000068438	309549
PAK3	X-linked mental retardation	IVS6+4A>G	5'ss	1	-0.3	ENSG00000077264	300304
BRCA2	Breast cancer predisposition	E16+188G>C	5'ss	1	-4.24	ENSG00000139618	113705
SEDL/TRAPPC2	X-linked spondyloepiphyseal dysplasia tarda	IVS4+1A>G	5'ss	2	-21.95	ENSG00000196459	313400
LDLR	Familial hypercholesterolemia	IVS12+2T>C	5'ss	2	-8.3	ENSG00000130164	144010
OPA1	Autosomal dominant optic atrophy	E20+166G>A	5'ss	2	-4.55	ENSG00000198836	165500
DMD	Muscular dystrophy	IVS32+5G>A	5'ss	1	-0.99	ENSG00000198947	310200
RPGR	Retinitis pigmentosa	IVS10+3A>G	5'ss	2	-2.32	ENSG00000156313	300029
WT1	Frasier syndrome	IVS9+5G>A	5'ss	0	-1.91	ENSG00000184937	136680
TG	Congenital hypothyroidism	IVS19+3_4delAT	5'ss	1	-1.44	ENSG00000042832	188450
PKD1	Autosomal dominant polycystic kidney disease	c.11156G>A	5'ss	0	-1.12	ENSG00000008710	173900
ADAR1	Dyschromatosis symmetrica hereditaria	c.1601G>A	5'ss	1	-0.14	ENSG00000160710	127400
BRCA2	Fanconi anemia	c.-40+1G>A	5'ss	1	-1.66	ENSG00000139618	610832
BRCA1	Early onset breast and ovarian cancer	c.441+2T>A	5'ss	2	-0.54	ENSG00000012048	113705
BRCA2	Early onset breast and ovarian cancer	c.8754+3G>C	5'ss	1	-1.02	ENSG00000139618	600185
BRCA1	Hereditary ovarian and breast cancer	c.560+2T>A	5'ss	2	-0.54	ENSG00000012048	113705
F9	Hemophilia B	c.252+3G>C	5'ss	2	-0.42	ENSG00000101981	306900
CSTB	Unverricht-Lundborg disease	c.66G>A	5'ss	1	-1.04	ENSG00000160213	254800
RASA1	Capillary malformation-arterovenous malformationo	c.2925+1G>T	5'ss	1	-0.69	ENSG00000145715	608354
IDUA	Mucopolysaccharidosis type 1	c.1727+3G>C	5'ss	1	-2.25	ENSG00000127415	252800
GLA	Fabry disease	c.801+2_801+3insT	5'ss	1	-2.41	ENSG00000102393	301500
