# Supplementary material for: Transposon clusters as substrates for aberrant splice-site activation
Source: RNA Biol. 2020 Sep 23;18(3):354–67. doi: 10.1080/15476286.2020.1805909 (PMC7951965; doi:10.1080/15476286.2020.1805909)
Supplement: Supplemental Material [file KRNB_A_1805909_SM0818.zip › Supplementary information/Table S2 rev.pdf]

Supplementary Table S2 Existing sense *Alu* exons that use 5'ss homologous to that activated in the *F8 Alu*J by mutation C>T at intron position +2

***Alu*J subfamily**	**Gene symbol**	**Gene product**	**Exon inclusion (%)**	**Accession number**	***Alu*J sequence over human transcripts***
*Alu*Jb	*CASQ2*	Cardiac calsequestrin 2	1/12 (8)	NM_001232	GGCCAGGTGCTGTGGCTCACACCTGTAATCCCAACACTTTGGGAGGCTGACGGGGGTGGATTGCATGAGCCTTGGAGTTGGAGACCAGCCTGGGCAACATGGTGAGACCCCATCTCTACAAAAAATACAAAAATTAGCTGGGTGTGGTGGCACACACTTGTAGTCCGAACTACTTGGGAGGCTTGAGGTGGGAGGATCACTTGAGCCCAGGAGGTTGAAGCTGCAGTGAGCTGTGATCATACCATTGTACTCCAACCTGGGCAATGGAGTGAGATCCTCTCTCAAAAAATAAAAATATAAAAAATAATAAA
*Alu*Jb	*ZNF532*	Zinc finger protein 532	3/36 (8)	NM_018181	GCACGGTGGCTCACACCTGTAGTCCCAGCACTTTGAGAGGCCAAGGCAGGAGGATGGCTTGAGGCCAGGAGTTTGAGAGCAGCCTTGACAATATGGTGAGACCCTGTCTGTACAAAAAAAAAAAAAGAACATTGGCTGAGTGTGGTGGCGTGTGCCTGTGGTCTCAGTTATTGGGGAGGCTGAGGAGTGAGGATTGCTTGAGGCCAGGAGGTGGAGTCTGCAGTGAGCTGTGATCACGCCACTGTACTCCAGCCTGGGCAACAAAGTGAGACACTATCTCAAAACAACAACAATAATAAAAAA
*Alu*Jo	*HNMT*	Histamine N-methyltransferase	12/112 (11)	NM_006895	GGCCAGATGTGTTGGTTCACGCCTGTAATCCTAGCACTTCGGGAGGCTGAGGCGGGAGGATTACTTGAGCCTAGGAGTTTGAGACCAGCCTGGGCAACATAGCAAGATCCCATCTCTACAAAAAAGTGAAAAAGTTAGCTGAACAAGGCGGCATGCACATGCTACTCCAGACGCTGAAGTGGGAAGATCACTTAAGTCCGAGAGATCGAGGCTTCAGTGAGATATGGCTGAGACACTGCTCTCAGCCTGGATGACAGAGTGAGAACCTGTCTCAAACAAGAGAAAAAAATAAA
*Alu*Jo	*MVD*	Mevalonate diphosphate decarboxylase	10/156 (6)	NM_002461	GGCGTGGGGCTCTCGCCTGTAATCCCAGTACTTAAGAGGCTGATGGGGGAGAAGCACTGTAGGCCAGGAGTTCAAGACCAGCCTGGGCAACATAGTGAGACCCCCCCCCATCTCTACAAAAAATAAAAAAAT
*Alu*Jo	*NUP62*	Nucleoporin, 62 kD	24/438 (5)	NM_153719	GGCTCACGCCTGTAGTCCTGGCACTTTGAGAGGACAAAGAGGGAGGATCGCTTGAGCCCAGGAGTTGAAGACCAGGGCAACAGAGTGAGATCCCATTTCCACAAAAAATAAAAAAAGATTATCCACGCATGGTGGCATGCACCTGTAGTCCTAGCTACTCTGGGAGGCTGAGGTGGGAGGATCACTTGGGTCCAGGAGGTTGAGGCTGCAGTGAGTTGTGATCATGTCACTGCATTCCAGCTAGTATGACAGAAAAAAACCCTTTCCCAAAAGAAAAAGAAA
*Alu*Sc	*STK11*	serine/threonine protein kinase 11	3/72 (4)	NM_000455	GGCCGGGCGCGGTGACTCACGAGAGCAAGAGATCCAGACCATCCTGGCCAACATGGTGAAACCCCATCTCTACTAAAAATACAAAAATTAGCTGGGCGTGGTTGCGGGCACCTGTAGTCCCAGCGACTCAGGGGCTGAGTCAGGAGAAATCGCTTGAACCCGGGAGGCAGAGGTTGCAGTGAGCTGAGATCATGCCATTGCACTCTGGCCTGGCGACAGAGTGAGACTCCGTCTCCAAAAAAAAAAAAAACAAGAAA
AluSq	*SLC35B3*	solute carrier family 35, member B3	2/29 (7)	NM_015948	GGCCAGGTGCAGTGGCTCACGCCTGTAATCCTAGCACTTTGGGAGGCCGAGGCAGGTTGATCACCTGAGGTTAGGAGTTCGAGACCAGCCTGGCCAACATGGTGAAACCCCGTCTCTACTAAAAATATAAAATTAGCTGGACATGGTGGCGGGCACTTGTAATCCTAGCTACTCAGGAGGTCAGGAGGCTGAGGCAGGAGAATGGTTTGAACCTGGGAGGCGGAGGTTGCAGTGAGCCAAGATGGCGCCACTGCACTCCAGCCTGGGCAACAAGAGCGAGACTCCGTCTCAAAAAAAAAAA
*Alu*Sq	*KYAT1 (CCBL1)*	cysteine conjugate-beta lyase; kynurenine aminotransferase	2/36 (6)	N.A.	AGCTGGGTATGGTGGCTCACGCCTATAATCCCAGCATTCTGGGAGGCTAAGGCTGGCAGATCACCTGAGGTCAGGAGTTCAAGACCAGCCTGCCCAACATGGTGAGATCCCATCTCTAATAAAAATTAGCTGGGCGTGGTGGTG

					GGTGCCTGTAATCCCAACTACTTGGGAGGCTGAGGCACAAGAATCGCTTGAACCCGGGAGGCGGAGGTTGCAGTGGGCCGAGGTGGTGCCATTGCACTCCAGCCTGGG
*Alu*Sq	*TRIM13 (RFP2)*	Tripartite motif containing gene 13	4/68 (6)	NM_001007278	GGCCGGGTGCAGTGGCTCAGGCCTGTAATCGCAGCACTTTGGGAGGCTGAGGTGGGCGGATCACCTGAGGTCAGGAGCTCGAGACCAGCCTGGCCAAAATG<u>GT</u>AAAACCCTGTCTCTACTAAAAATACAAAAAATTAGCTGGGCATGGTGGCAAGTGCCTGTAATACCAGCTACTTGGGAGGCTAAGGCAGGAGAATCACTTGAACCCAGGAGGCGGAGGTTCCAGTGAGCCAAGATCACACCATTGCACTCCAGCCTGGGTGACAAGAGCAAAACTCTGTCCCAAAAAAAAAAAAAAAAA
*Alu*Sx	*C1orf109 (FLJ20508)*	Uncharacterized protein C1orf109	29/37 (78)	NM_017850	GGCCGGGCGCGGTGGCTCACGCCTGTAATCCCAGCACTTTGGAAGACCGAGGCGGGCGGATCACCTGAG<u>GT</u>CAGGAACCCCGTCTACTAAAAATACAAAAATTAGCCAGGCGTGTTGGCGGGCACCTGTATTCCCAGCTACTCGGGAGGCTGAGGCAGGAGAATTGCTTGAACCCGGGATGCGGAGGTTGCAGTGAGCCGAGATAGCGCCACTGCACTCCAGCCTGGGCGACAGTGAGACTCCGTCTCGAAAAAAAAACAAAAAAAAAAA
*Alu*Sx	*PSEN1*	presenilin 1	1/57 (2)	NM_000021	GGCTGGGCACAGTGGCTCACACCTGTAATCTGCCAGCACTTTGGGAGGCCGAGGCAGGTAGATCACCTGAGGTCAGGAGTTCGAGACCAGCCTCGCCAAAATG<u>GT</u>GAGACCCTGTGTCTACTAAAAATACAAAAAATTAGCCGGGTGTGTTGGCGTGTACCTGTTATCCCAGCTACTCGGGAGGTGAGGCAGGAGAATCGCTTGAACCCAGAAGGTGGAGGTTGCAGTGAGCCGAGATCATGCCACTGCACTCCAGACTGGGCTACAGAGCGAGACTCCATAAGAAAAAAAGAAAAAAAAA
*Alu*Sx	*MED1 (PPARBP)*	peroxisome proliferator-activated receptor-binding protein	2/68 (3)	NM_004774	GGCTGGGCGTGGTGGTTCACATCTGTAATCCCAGCACTTTGGGAGGCTGAGGCGGGTCGATCACCTGAGGTCAGGAGTTCGAGACCAGGCTGACCAACAAG<u>GT</u>AAAACCCTGTCTCTACTAAAAAGACAAAATTAGCTGGGCATAGTGGCAGGCGCCTGTAGTCCCAGCTACTCGGGAGGCTGAGACAGCAGAACCGCTTGAACCCGGGAGGTGGAGGTTGCAGTGAGCCAAGATCATGCTACTGCACTCCAGTCTGGGCATCGGAGCGAGACTCCATCTCAAAAAAAAAAAAA
*Alu*Sx	*HAUS1 (CCDC5)*	HAUS augmin like complex subunit 1; coiled-coil domain containing, 5	2/131 (2)	NM_138443	GGCCAGGTGCAGTGGCTCACGCCTGTAATCCCAGCACTTTGGGAGGCCAAGGTGGGCGGATCACTTGAGACCAGGAGTTTGAGACAAGCCTGGCCAAGATG<u>GT</u>AAAACCCCGTCTCTACTAAAAATACAAAAATTAGCCAGGTGTGGTGGTATGTGCCTGTAATCCCAGCTACTTGGGAGGCTGAGGCGGGAGAAATTCTTGAACCCGGGAGGTGGAGGCTGAAGTGAGCAGATATCACACCACTGCACTCCAGCCTGGGCAACAGAGTGAGACTCTCTCAACAACAAAAAAA
*Alu*Y	*CCDC74A*	Coiled-coil domain containing 74A	3/39 (8)	NM_138770	CCGGGCGCGGCGGCTCACGCCTGTAATTCCAGCACTTTGGGAGGCCGAGACGGGCGGATCACGAGGTCAGGAGATCGAGACCATCCTGGCTAACACG<u>GT</u>GAAATCTCGTCTCTACTAAAAATACAAAACAATTAGCTAGGCGTGGTGGTGGGCGTCTGTAGTCCCAGCTACTCGGGAGGCTGAAGCAGGAGAATGGCGTGAACCCGGGAGGCGGAGCTTGCAGTGAGCCGAGATCTTGCGACTGCACTCCAGCCTCGGTGACAGAGCGAGACTCCGTCTCAAAAAAACAAAAACAAAAA
*Alu*Y	*FAM13B (C5orf5)*	Family with similarity 13, member B; chromosome 5 open reading frame 5	3/28 (11)	NM_016603	GGCCGGGTGCGGTGGCTAACGCCTGTAATCCCAGCACTTTGGGGGCCGAGACGGGCAGATCACGAGGTCAGGAGATGGAGACCATCCTGGCTAACACG<u>GT</u>GAAACCCCATCTCTACTAAAAATACAAAAAAATTAGCCGGGCGTGGTGGCAGGCACCTGTAGTCCCAGCTACTTGGGAGGCTGAGGCAGGAGAATGGCATGAACCTAGGAGGCAGAGCTTGCAGTGAGCCGAGATCATGCCACTGCACTGCAGCCTGGGCAACAGAGCGAGACTCCGTCTCAAAAAAAAAAAAAAA
*Alu*Y	*WBP2*	WW domain binding protein 2	6/1640 (0.4)	NM_012478	GGCCGGGCGCAGTGGCTCACGCCTGTAATCCCTGCACTTTGGGAGGCCGAGGCAGGTGGATCACGAAGTCAGGAGATCGAGACCATCCTGGCTAACACG<u>GT</u>GAAACCCCGTCTCTACTAAAAATACAAAAAATTAGCCAGTCGT

					GGTGGCGGGCGCCTGTAGTCCCAGCTACTTGGGAGA CTGAGGCAGGAGAATGGCATGAACCCAGGAGGCGG AGCTTGCAGTGAGCCGAGATCACGCCACTGCACGCC AGCCTGGGTGACAGAGTGAGACTCCGTCTCAAAAAA AAAAAAAAAAAAATAAAGAAAA
*Alu*Y	*UBE2L3*	ubiquitin-conjugating enzyme E2 L3	1/555 (0.2)	NM_003347	GCCAGGTGCAGTGGCTCACGCCTGTAATCCAGCACT TTGGGAGGCGGAGGCAGGTGGATCACGAGGTCAGG AGATCGAGACCATCCTGGCTAACACGGTGAAACCCC GTCTCTACTAAAAAATTACAGAAAATTAGCCGGGCA TGGTGGCAGGTGCCTGTAATCCCACTACCCGGGAGG CTGAGGCAGGAGAATGGCATGAGCCTGGGAGGCGG AGCTTGCAGTGAGCCGAGATTGTGAGCTGTGATTGT GCCACTAACTCAGCCTGGGCGACAGAGCGAGACTCC ATCTCAAAAAA
FLAM_A	*NSE2 (FLJ32440)*	SMC5-SMC6 complex SUMO ligase	3/52 (6)	NM_173685	GCTAGGTGTGGTGGCGCACGCCTGTAGTTCCAGCTA CTTGGGAGGCTGAAGTGGGAGGATCACTTGAGCCCA GGAGTTCAAGATCAGCCTGGACAACATAGTAAGACT CCATCTCTTTAAAAAAAAAAAA
FLAM_C	*CHKB*	choline kinase beta	20/59 (34)	NM_005198	GCCTGTATTCCCAGAGCTTTGGGAGGCTGAGGCGAG AGGATCACTTGAGCACAGGAGTTCGAGACCAGCCTG GACAACATAGTGAGACCCCCATCTCTAAATAAAAA
FLAM_C	*PPA2*	inorganic pyrophosphatase 2	3/129 (2)	NM_006903	CTGGGTGCAGTGGCTCATGCCTGTAATCCCAGCACTT TGGGAGGCGAGCTAAGAGGATCTCCTGAACCCAAGA GTTCAAGACCAGCCTGGGCAACATGGTAAGACTGAG TCTCAAAAAAATAATAATAAA

Table data were compiled from TranspoGene [1]. *Sense *Alu* sequences that overlap exons are in black; their remaining portions are in red; the 5'ss GT dinucleotides are underlined. Their alignments are in Fig. S3.

Reference:

1. Levy A, Sela N, Ast G. TranspoGene and microTranspoGene: transposed elements influence on the transcriptome of seven vertebrates and invertebrates. Nucleic Acids Res 2008; 36:D47-52.
