# Supplementary material for: Transposon clusters as substrates for aberrant splice-site activation
Source: RNA Biol. 2020 Sep 23;18(3):354–67. doi: 10.1080/15476286.2020.1805909 (PMC7951965; doi:10.1080/15476286.2020.1805909)
Supplement: Supplemental Material [file KRNB_A_1805909_SM0818.zip › Supplementary information/Table S3 rev.pdf]

Table S3 Homologs of SHAPE-predicted *F8* apical triloop in existing sense *Alu* exons have largely enhancer activities

**Gene with sense *Alu*-derived exon**	**Triloop context[*]**	**Average ESRseq score[**] of 4 overlapping hexamers**
CASQ2	GUUGGAGAC	0.39
ZNF532	GUUUGAGAG	-0.22
HNMT	CACUGCUCU	0.14
MVD	GUUCAAGAC	0.41
NUP62	GUUGAAGAC	0.41
STK11	GAUCCAGAC	0.08
SLC35B3	GUUCGAGAC	0.46
KYAT1 (CCBL1)	GUUCAAGAC	0.41
TRIM13 (RFP2)	GCUCGAGAC	0.39
C1orf109 (FLJ20508)	GACCGAGGC	0.46
PSEN1	GUUCGAGAC	0.46
MED1 (PPARBP)	GUUCGAGAC	0.46
HAUS1 (CCDC5)	GUUUGAGAC	0.08
CCDC74A	GAUCGAGAC	0.49
FAM13B (C5orf5)	GAUGGAGAC	0.47
WBP2	GAUCGAGAC	0.49
UBE2L3	GAUCGAGAC	0.49
NSE2 (FLJ32440)	GUUCAAGAU	0.40
CHKB	GUUCGAGAC	0.46
PPA2	GUUCAAGAC	0.41
PKP2	GUUCGAGAC	0.46
	Mean (±SD)	0.36 (±0.19)

[*]*F8 Alu*J triloop homologs in existing *Alu*-derived exons are underlined. Their alignment is shown in Fig. S3. [**]ESRseq scores were as defined by Ke and co-workers [1]. Positive score values indicate splicing activators [1].

Reference

1. Ke S, Shang S, Kalachikov SM, Morozova I, Yu L, Russo JJ, et al. Quantitative evaluation of all hexamers as exonic splicing elements. Genome Res 2011; 21:doi10.1101/gr.119628.110.
